# The research of touch screen usability in civil aircraft cockpit

**DOI:** 10.1371/journal.pone.0292849

**Published:** 2024-02-08

**Authors:** Xiaoli Wang, Wei Guo, Zhenwei Zhong, Rui Zeng, Jiong Zhang, Lijing Wang

**Affiliations:** 1 Demonstration Center of Future Product, Beijing Aircraft Technology Research Institute, COMAC, Beijing, China; 2 Institute of Future Technology Research, Beijing Aircraft Technology Research Institute, COMAC, Beijing, China; 3 School of Aeronautic Science and Engineering, Beihang University, Beijing, China; Sunway University, MALAYSIA

## Abstract

With the advancement of touch screen technology, the application of touch screens in civil aircraft cockpits has become increasingly popular. However, further analysis and research are required to fully promote its applications. The paper researched the usability of touch screens in aircraft cockpit considering the operation performance and subjective NASA-TLX workload evaluation, conducted experimental research on three touch gestures: click, drag, and zoom. Additionally, a comparative analysis was conducted on the touch performance under different layouts, positions, touch sizes, dragging direction angles, and zoom multiples. The touch performance indicators include operation time, error rate, operation speed, and workload. The experimental results show that the 21 mm size has the minimum operation time and workload, and 18 mm size has the lowest error rate in the clicking tasks. Additionally, the performance and workload of the captain’s layout are better than those of the co-pilot’s layout, and the performance of the center console position is best. The operation speed of the dragging tasks is faster when performed at position R3 compared to other positions. The dragging moving angles with better operation speed are 80°-190° and 250°-290°. The operation performance and workload of the zooming tasks vary depending on the zoom multiples. As the multiple increases, the operation time and workload also increase. There is no difference in operation performance or workload between zooming in and zooming out. The paper provides experimental support and suggestions based on human operation and subjective NASA-TLX workload evaluation for the application of touch screens in civil aircraft cockpits.

## 1. Introduction

### 1.1 Background

The touch screen integrates display and control in a natural interactive manner, greatly simplifying the operation of the human-machine interface. The touch technology has been used in various fields, such as mobile phones, electrical equipment, and automobiles. And the touch technology has also been gradually introduced into the aircraft cockpit. The first application in aviation is the F-35 aircraft of the US military. Since then, Garmin, Thales, Gulfstream, Honeywell, and other companies have also conducted lots of research works on touch screens in aircraft cockpits, for example, Garmin’s G3000 [1] and G5000 [2] navigation systems with touch operation, the G3X touchable avionics system, ODICIS [3] cockpit with one touch screen of Thales, and the Primus Epic [4] aviation system designed by Honeywell. In business jets, a touch-operated avionics systems are used in Gulfstream’s G500/600. The latest applications in the cockpit of civil aircraft are A350 and B777-X [5], where touch control is used as a backup operation. In addition, there are some guidelines or standards regarding touch screens in aircraft, such as AC 120-76D [6], AC 20–175 [7] and SAE ARP60494 [8].

### 1.2 Research significance

In addition, there are some academic studies on the application of touch screens in aircraft cockpits. Some researchers have compared and analyzed the touch screens with traditional devices, such as MCP(Mode Control Panel), MCDU(Multi-Control and Display Unit), cursor, and keyboard [9, 10]. Those studies show that there are many advantages to using the touch screens in the cockpit, the operation performance and workload of pilots have both improved [11]. However, there are also differences in various layouts and positions. For instance, the advantages of the overhead panel and glare-shield are smaller than those of the front instrument panel and center console [12, 13]. The layout and position of the touch screen in the cockpit are the main limiting factors for its application. And some studies have also considered about the touch control methods [14], vibration environment in the cockpit [15], G-Force [16], hand support [17], etc. Although the application of touch screens in the cockpit of civil aircraft has attracted more and more attention and conducted a lot researches, the practical application of touch screen control in the cockpit is still a challenge. Kaminani et al. [1] discussed the advantages and challenges that the touch screen technology has brought to the flight deck from a human factors perspective. Some issues have not been resolved yet. A lot of research works have confirmed the prospects of the application of touch screens in the cockpit. Touch screen operations are gradually being introduced to aviation. The touch screen integrates display and control, which is more intuitive and convenient than commonly used mouse and cursor operations [1]. In order to further promote the application of touch screens in the cockpit, we need to first determine where and how the touch screens are used. So, the paper will explore the usability of touch screens in the cockpit based on that issue.

### 1.3 Touch control gestures and variable

The common touch gestures include clicking, dragging, and zooming. Many scholars have conducted research on these gestures in various application fields. For example, Kim et al. [18] had studied the touch control of click, swipe, and zoom in the vehicle information system. Annie et al. [19] found that a simple click operation with a touch screen is good, but it is poor for continuous adjustment and list scrolling. And Bjørneseth et al. [20] found that the button operation of a touch screen is faster and had fewer errors than the gesture operation. In addition, Gao et al. [21] had also carried out a research on the touch gestures. Although many researches have been done, the application fields are completely different, and the results are also correspondingly different. And the operation of clicking is a promising direction of touch screens in the cockpit. Whether in normal flight or in abnormal flight conditions, pilot can perform certain operations by clicking, such as checking the electronic checklist, selecting a system page, viewing the flight plan information, adjusting display configuration, selecting the items of menu bar, and controlling other functions that have minimal impact on flight safety [10, 12, 22]. Additionally, pilots can also use drag and zoom functions to navigate the map, flip through system pages, and check terrain and weather radar. But it is also necessary to maximize the efficiency and reduce the operation errors of touch operations. So, the paper conducted a series of experiments and analysis on touch gestures in the aircraft cockpit environment.

The application of touch screens in the cockpit should not only consider how to operate them, but also where to use them and what sizes are suitable. Target size of the touch is a critical factor in touch operation. Parhi et al. [23] found that the best performance value was achieved when the target size reached 9.2 mm on a small touch device. Increasing the target size did not have any effect on the subjective feeling or error rate. Park et al. [24] found that the target sizes of 7 mm and 10 mm were sufficient for touch operation on a mobile phone. Kim et al. conducted a research on the button sizes of touch screens for vehicle information systems. The study showed that when the button size is less than 22.5 mm, both the operation performance and workload are affected. However, there was no further change once the button size exceeded 22.5 mm. While Colle et al. [25] conducted a study on the target size of the shared phone booth and found that 20 mm is a watershed. The results varied in different environments, so further experimental researches should be conducted with the cockpit application.

Different layouts or positions also obviously affect performance. For example, Park found that the transition time in the center area is shorter than that in the edge area, and the error rate on the left side is lower. Dodd conducted a study on the effects of flight turbulence, cockpit display location, and size on touch performance and workload for pilots. The layout of civil cockpits is relatively complex, and there are many position divisions such as the overhead panel, glare shield, front instrument panel, and consoles. Each layout and positon has unique operation requirements and displays information. The effects of touch screens in different positions are also varied. For example, Zhang Yanwen et al. found that the operation time for touch screens at the overhead panel position is longer than that for traditional operation. Due to the overhead panel being located above the pilot’s head, its readability is poor and its operation is inconvenient. However, there are other positions, such as the front instrument panel and center console area, that are located in front or to the side of the pilot, and they are relatively easy to operate. So, the layout and position of the touch screen application should be analyzed and evaluated based on the cockpit operation performance.

### 1.4 Scope

The usability of touch control is a crucial area of research in cockpit environment, aimed to promote the application of the touch screens in aircraft. The paper conducts experimental research on touch gestures, specifically click, drag, and zoom, based on human operation in civil aircraft cockpits The study also considers various factors that may affect the performance, including different layouts, positions, sizes, moving directions, and zoom in/out multiples. The objective is to evaluate the operational performance and workload associated with these factors. The NASA-TLX scale is used to evaluate the workload of touch operations. It utilizes six dimensions to assess the workload, including mental demand, physical demand, temporal demand, overall performance, effort, and frustration level. The paper conducts a fundamental study on the application of touch screens in the cockpit. Based on the analysis of experimental data, it is possible to determine the layout or position, click size range, drag direction angle range, and zoom display form of touch control that offer good operational performance. The paper provides experimental support and suggestions for the application of touch screen in the aircraft cockpit, based on human operation and subjective NASA-TLX workload evaluation.

## 2. Experiment method

### 2.1 Subject

Fourteen participants participated in the experiments, all of whom were male college students from Beihang University aged 20 to 30 years old(ME = 25.4, SD = 1.74). The average height of the participants was 175 cm, and they were all right-handed. All participants voluntarily participated in the experiment, and they all possess some knowledge of aviation.

### 2.2 Experiment environment

**Fig 1:**
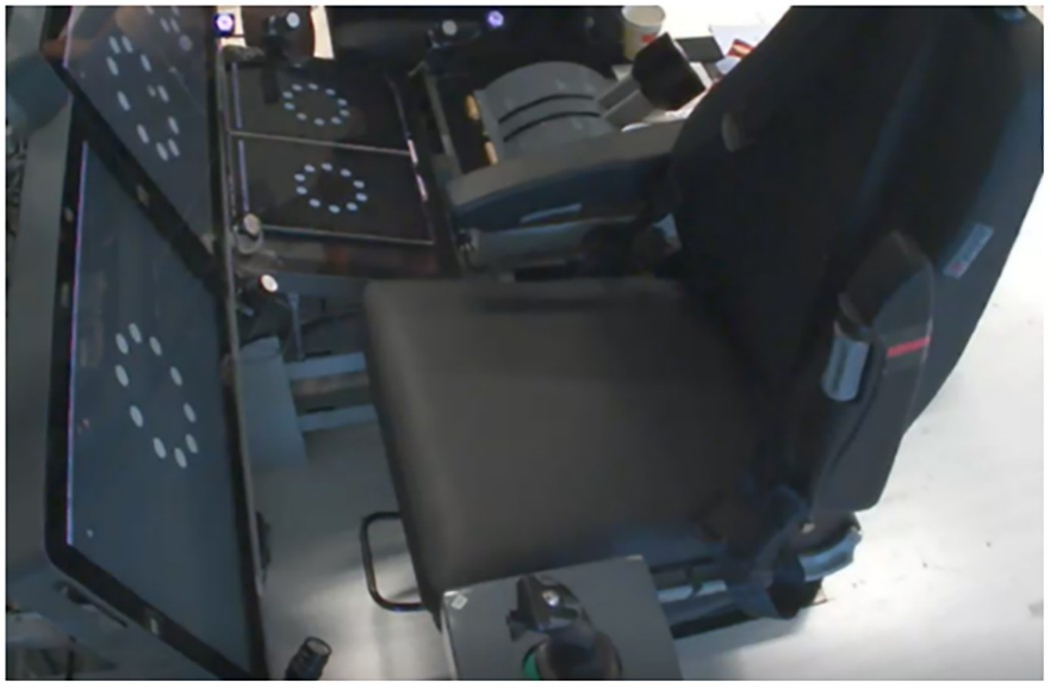
Touch screen experiment environment.

The research team constructed an experimental cockpit platform and touch equipment based on the characteristics of the civil aircraft. This included the main components such as the front instrument panel, center console, and seats. The layout, position, and physical sizes of the civil cockpit are similar to those of the A320. In the experiments, the display screens in the front instrument and center console area have been replaced with touch screens based on that cockpit, as shown in Fig 1. The Microsoft surface go tablets are used in L3 and R3 positions, and the touch screens in other positions are customized devices.

**Fig 2:**
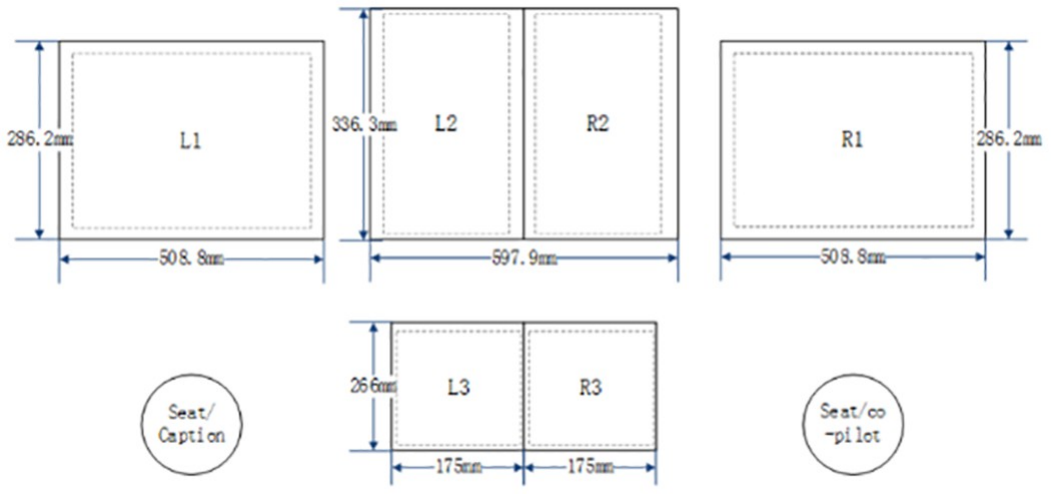
Schematic diagram of touch screen layouts and sizes.

In the experiments, the front instrument panel area consists of three touch screens. The left (L1) and right (R1) positions of the front instrument panel are identical, measuring 508.8 mm x 286.2 mm. The middle position of the front instrument is divided into two display area, L2 and R2, measuring 597.9 mm x 336.3 mm. The central console position (L3, R3) consists of two touch screens, each measuring 266 mm x 175 mm, as shown in Fig 2.

### 2.3 Experiment design

In order to study the usability of touch control in the cockpit environment based on human operation, the paper conducted experiments on three control gestures: click, drag, and zoom. First, we analyzed the normal flight missions and the display interface of the cockpit. We assessed whether it is feasible to replace cursor operation with touch operation in the primary flight display/navigation display interface, system display interface, and flight management page. Additionally, we determined the most suitable type of touch operation. Then, three types of experimental tasks are designed in different positions where touch operations are likely to be used. Finally, we determined the independent variable factors: touch sizes, touch space layouts, positions, operation movement directions, and zoom in/out multiples. The experimental tasks in the paper involve basic touch operations such as simple clicks, drags, and zooms. The experiments do not specifically address cockpit interface design elements or flight operation tasks. But in the experimental interface, the commonly used black background and gray design of the cockpit are used. Detail designs are in section 2.3.1–2.3.3. Before conducting the official experiments, designers carried out some preliminary experiments to select appropriate variable values. For the click experiment, we selected sizes ranging from 9mm to 21mm with intervals of 1mm and 3mm, and conducted separate tests. The designers recorded and analyzed the experimental data, and the results were used to select the appropriate size interval. In the zoom experiment, zoom multiples ranging from 2 to 6 were also tested to determine the optimal experiment multiples. In addition, the tasks in experiments are all simple clicks, drags and zooms tasks. Participants who have a good understanding of aviation knowledge and are familiar with touch screen operations can successfully complete each experimental task. It is not necessary to have professional aviation skills to operate the tasks. The experimental details are as follows.

#### 2.3.1 Click experiment

In the click experiments, the paper selected nine target sizes: 9 mm, 10 mm, 11mm, 12 mm, 13 mm, 14 mm, 15 mm, 18 mm, and 21 mm. The experiments are distributed on two layouts: captain seat area and the co-pilot seat area, as shown in Fig 2. Additionally, there are three positions under each layout: left/right area of the front instrument panel and the central console, which are L1, L2, R2, R1, L3, and R3. The combination relationships between various variables in the experiments are shown in Table 1.

**Table 1:** The independent variable levels and variable combinations in click experiments.

Layout	Position	Target Size/mm								
**L(captain)**	L1	9	10	11	12	13	14	15	18	21
	L2	9	10	11	12	13	14	15	18	21
	L3	9	10	11	12	13	14	15	18	21
**R(co-pilot)**	R1	9	10	11	12	13	14	15	18	21
	R2	9	10	11	12	13	14	15	18	21
	R3	9	10	11	12	13	14	15	18	21

**Fig 3:**
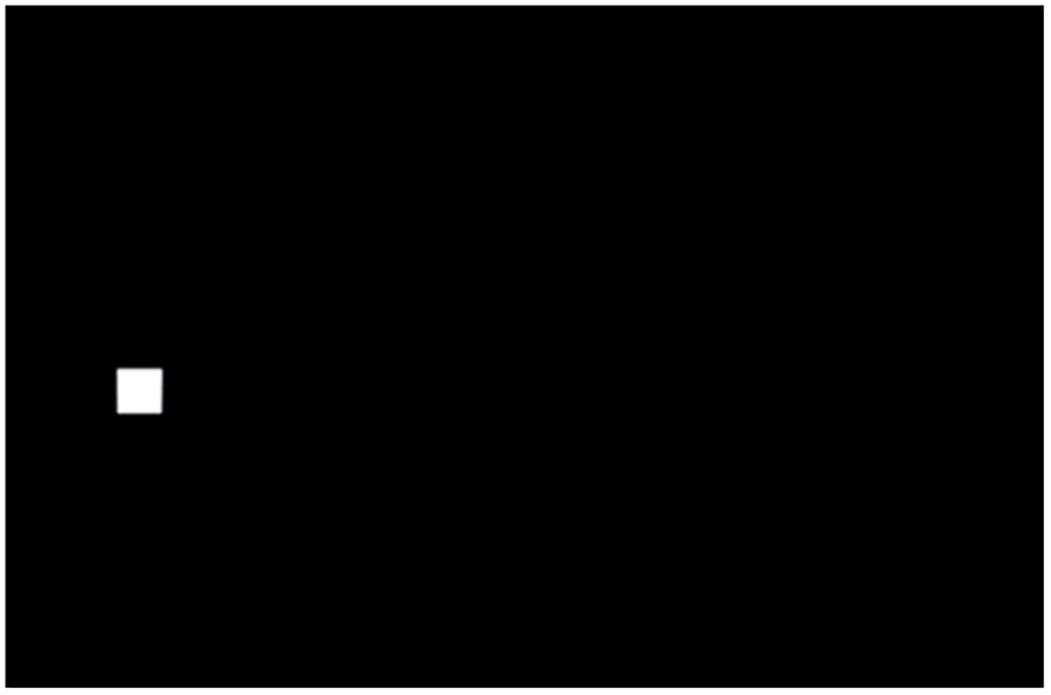
Click experiment interface.

Record the participants’ operation times, click errors and click positions in experiments. And participants evaluated the workload of each layout and size based on the NASA-TLX scale (see section 2.5 for details). The click experiment interface is shown in Fig 3. The experiment divides the screen into many squares based on the target size. For instance, in the experiment with 21 mm size, the entire touch screen is divided into a grid of 21 mm x 21 mm blocks in the interface, ensuring that there is no overlap between the blocks. When the experiment starts, square blocks of the set size appear at random positions on the interface. The participant needs to click and the next random square block appears, the experiment ends when all the square blocks on the screen have appeared. Each square block can remain visible for a maximum of 2 seconds, and it will automatically disappear and the next square block will appear if the participant does not click after 2 seconds. The participants have sufficient reaction time to complete the single click task within 2 seconds, which is a suitable time, and that limit with 2 seconds will not affect the results of the experiment.

#### 2.3.2 Drag experiment

In the dragging experiments, the paper mainly analyzed the effects of different dragging direction angles on touch operation. The distribution areas for the dragging experiments are L1, R1, L3, and R3. The specific experimental settings are shown in Table 2.

**Table 2:** The independent variable levels and variable combinations in dragging experiments.

Layout	Position
**L(captain)**	L1
	L3
**R(co-pilot)**	R1
	R3

**Fig 4:**
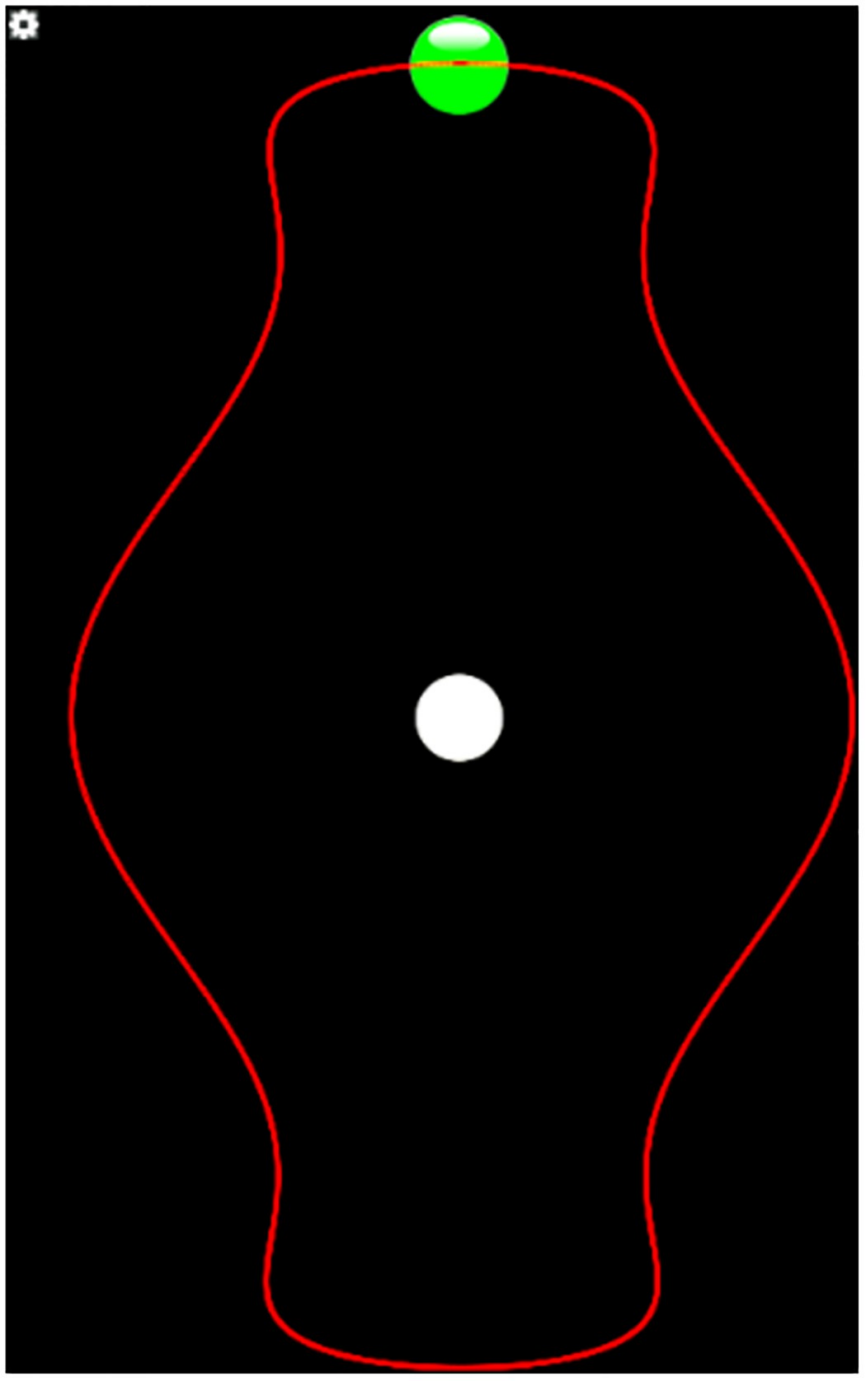
Dragging experiment interface.

The dragging experiment involves ten different shapes for dragging, a random dragging shape appears when the experiment starts, the participant drags the green ball along the given curve paths with different cambers in order to achieve a 360° closed-loop dragging (as shown in Fig 4, with other interfaces are shown in Appendix Fig 1 in S1 Appendix). Once an experiment is completed, the participant clicks the gray ball and another shape will randomly appear, and the participant will repeat the above operation. Record the participants’ operation times and positions in experiments. Participants evaluated the workload of each layout based on the NASA-TLX scale.

#### 2.3.3 Zoom experiment

The zoom experiment includes zoom in and zoom out experiments at various multiples (2, 3, 4, 5, and 6 times) and different display modes (displaying multiples values in real time or after releasing). The distribution areas for the zoom experiment are L1, L3, R1, and R3. The specific experimental settings are shown in Table 3.

**Table 3:** The independent variable levels and variable combinations in zoom experiments.

Layout	Position	Zoom	Display	Multiple				
**L(captain)**	L1	Zoom In	Real Time	2	3	4	5	6
			Not Real Time	2	3	4	5	6
		Zoom Out	Real Time	2	3	4	5	6
			Not Real Time	2	3	4	5	6
	L3	Zoom In	Real Time	2	3	4	5	6
			Not Real Time	2	3	4	5	6
		Zoom Out	Real Time	2	3	4	5	6
			Not Real Time	2	3	4	5	6
**R(co-pilot)**	L1	Zoom In	Real Time	2	3	4	5	6
			Not Real Time	2	3	4	5	6
		Zoom Out	Real Time	2	3	4	5	6
			Not Real Time	2	3	4	5	6
	L3	Zoom In	Real Time	2	3	4	5	6
			Not Real Time	2	3	4	5	6
		Zoom Out	Real Time	2	3	4	5	6
			Not Real Time	2	3	4	5	6

**Fig 5:**
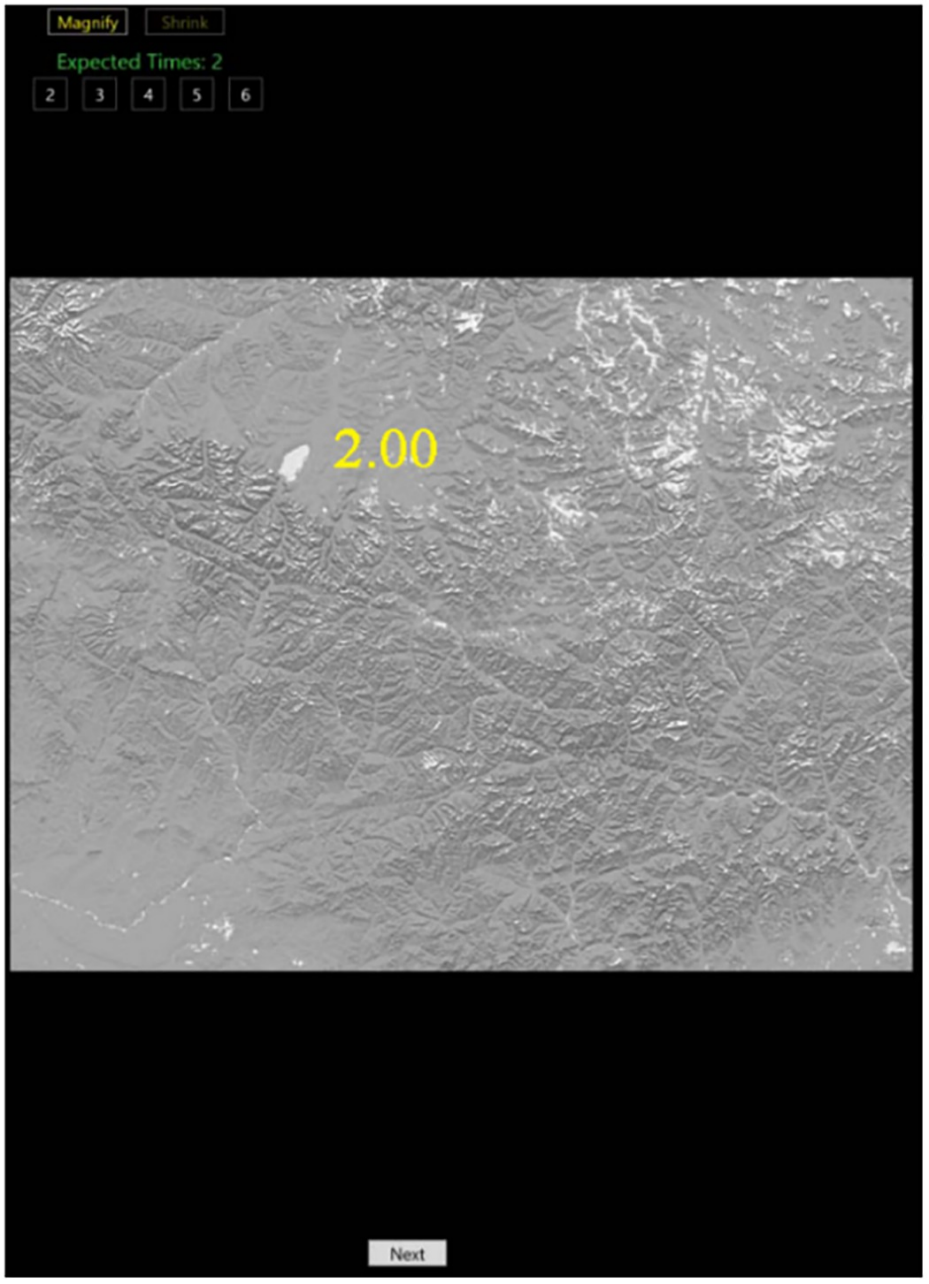
Zoom experiment interface(The map in figure is similar but not identical to the original image and is for illustrative purposes only, the map in figure is from USGS National Map Viewer (public domain): http://viewer.nationalmap.gov/viewer/).

In the experiment, the participant is required to zoom in or out the map based on the specified multiples and keep the actual operation error within the range of ± 0.1. In a single variable experiment, there are 10 map zoom tasks. Each task is completed under a specific multiple. After completing one zoom task, the user clicks "next" and another zoom task appears. This operation is repeated until all 10 tasks are completed. The zoom experiment interface is shown in Fig 5 (only a partial interface is shown for illustrative purposes). Record the participants’ operation times and zoom multiple values in experiments. And participants evaluated the workload of each layout, zoom, display, and multiple based on the NASA-TLX scale.

### 2.4 Experiment procedure

a) Firstly, inform the fourteen participants about the detailed experimental content and process. Conduct testing procedures before the formal experiment to familiarize them with the experimental process and also test the comfort through arm length and extension; all participants are instructed to complete the experiments accurately and quickly;
b) After testing, the formal experiments begin, the experiments are conducted in a random sequence, involving clicking, dragging, and zooming, respectively;
c) Each type of experiment is conducted separately, and the experimental sequences are also randomized. For example, in the click experiment, participants are randomly assigned to start the experiment in either the captain or co-pilot layout. Under each layout, different positions and sizes are combined to create random combinations, that take into account all the variables. The random combinations are used to conduct experiments. As well as the experiments of dragging and zooming. And at least one person supervises the experimental process;
d) In each experimental task, the participants open the experimental program on each touch screen. They then set the experimental variables, click “start”, and the experiment begins. Participants complete the task according to the experimental requirements and repeat the above steps to complete the next experimental task;
e) During the experiment, participants need to take appropriate breaks. For example, in the click experiment, participants are required to rest for 5 minutes after every 5 groups of experiments. After completing an experiment on one layout, participants are required to rest for 10 minutes. Participants are also required to rest for 20 minutes after completing the click experiment and workload evaluation. Additionally, participants can take breaks as long as they are needed, except during the experimental process. After the break, the participants are asked to confirm whether they can proceed to the next experiment. As well as the experiments of dragging and zooming;
f) After completing one type of experiment in either the captain’s or co-pilot’s areas, the participants are required to fill out a pre-prepared NASA-TLX workload assessment questionnaire to evaluate the workload. Fourteen participants participated in all experiments and completed subjective questionnaire evaluations.

### 2.5 Dependent variable

There are two categories of dependent variables: operation performance and workload. The former includes operation times, error rates, accuracy deviations, and operation speeds. The latter is evaluated using the NASA-TLX scale. The detailed information for each experimental dependent variable is shown in Table 4.

**Table 4:** The dependent variables and definitions in each experiment.

Experiment	Dependent Variables	Unit	Variables definition and Measure methods
**Click Experiment**	Operation Time	ms	The operation time refers to the time required for the operator to click on the square block.
	Error Rate	dimensionless	The error rate refers to the probability that the operator will not successfully click on the square block. It is obtained by calculating the ratio of the number of non-clicked square blocks to all appearing square blocks.
	Accuracy Deviation	mm	The accuracy deviation refers to the deviation between the position of the participant’s finger click and the center position of the target. The higher the accuracy deviation, the lower the accuracy.
	Workload	dimensionless	The workload is evaluated using the NASA-TLX.
**Drag Experiment**	Operation Speed	mm/s	The operation speed refers to the instantaneous speed at which the operator drags the ball. It is obtained by calculating the ratio of the distance of movement at each angle to the time of movement.
	Accuracy Deviation	mm	The accuracy deviation refers to the minimum deviation between the position of the operator moving the ball and the expected position in the shape.
	Workload	dimensionless	The workload is evaluated using the NASA-TLX.
**Zoom Experiment**	Operation Time	ms	The operation time refers to the total time used for the zoom in/out operation to achieve the given multiple.
	Accuracy Deviation	dimensionless	The accuracy deviation refers to the differences between the operator’s final results and the actual given zoom multiple as a percentage of the given zoom multiple. The higher the accuracy deviation, the lower the accuracy.
	Workload	dimensionless	The workload is evaluated using the NASA-TLX.

The NASA-TLX scale [26] is a commonly used method for measuring workload. It utilizes six dimensions to evaluate the workload: mental demand, physical demand, temporal demand, overall performance, effort, and frustration level. The mental demand refers to the level of mental effort required to complete a task; physical demand refers to the level of physical energy required to complete a task; temporal demand refers to the urgency of completing tasks; overall performance refers to the degree of satisfaction with self-performance when completing tasks; effort refers to the level of effort (physiological and psychological) required to demonstrate one’s own performance when completing a task; frustration level refers to the level of frustration, complacency, anger, and tension during the completion of a task. And each dimension is scored from 1–10 point, with 10 points corresponding to an extremely high workload and 1 point corresponding to an extremely low workload. And a lower workload is better than a higher workload. The total workload value of the participant is calculated using the weighted average method based on the six dimensions.

The experimental data has been checked for missing values and any data that did not meet with 3σ principle was eliminated. Afterward, statistical analysis was conducted. The repeated measures ANOVA analyses were conducted on each experimental independent variable and its corresponding dependent variable to analyze the relationship them in the experiments. At the same time, the normality was tested, the sphericity test was conducted, and the main effects of each variable were analyzed. The variables that did not meet the sphericity test were adjusted using the Greenhouse-Geisser method. And some p-values were adjusted using the Benjamini/Hochberg (B/H) method to control the false discovery rate (FDR) [27]. An association was considered to be statistically significant if its corresponding B/H-adjusted p-value was below 0.05, which corresponds to a FDR of 5%. And we have also conducted pairwise tests and adjusted it using the Bonferroni method.

The ethics approval had been granted by research institution’s ethics committee before conducting the experiment. All participants who participated in the experiment were required to sign the consent form prior to the experiment. Participants were also informed that they had the right to terminate and withdraw from the experiment at any stage, even after the data collection phase. All relevant ethical safeguards have been met with regard to participant protection.

## 3. Experiment result

### 3.1 Click experiment

**Fig 6:**
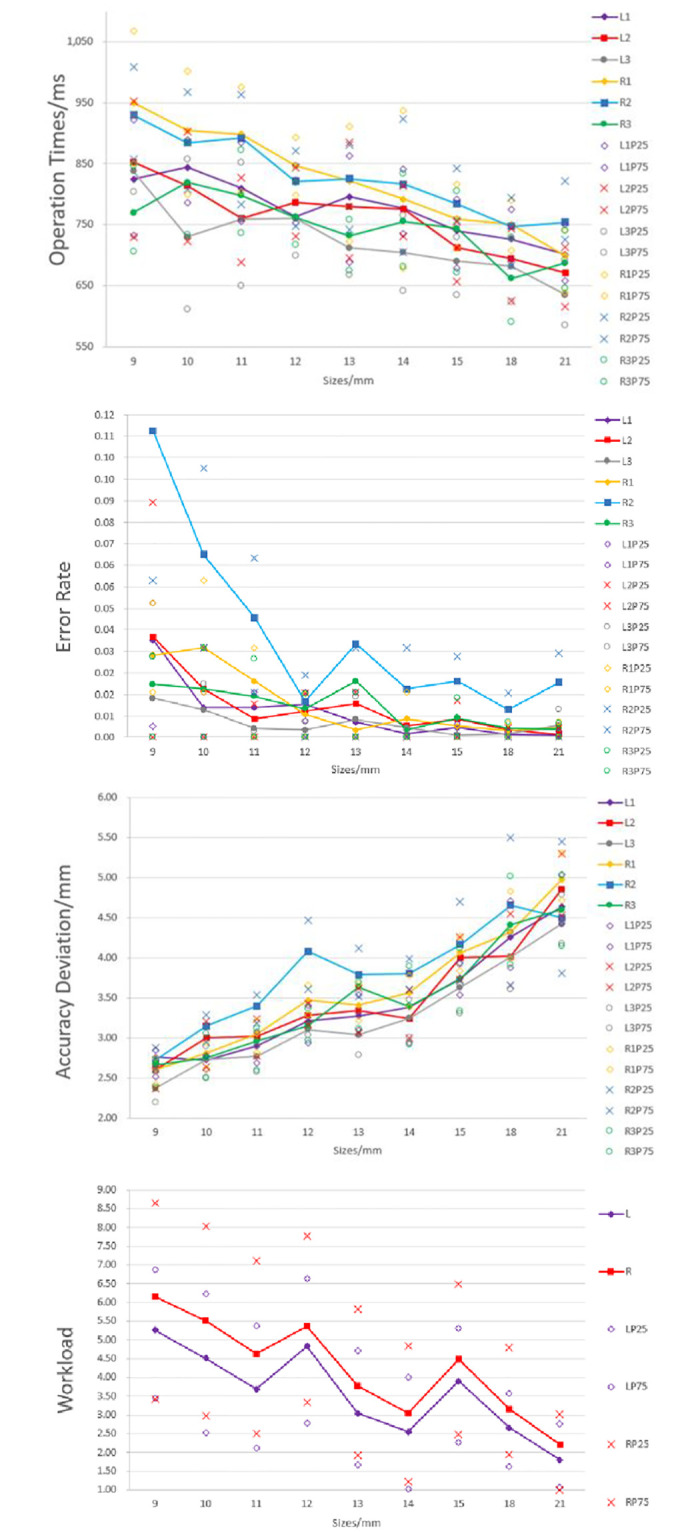
Effects of sizes on operation performance (operation time, error rate, accuracy) and workload in click experiments.

First, the paper analyzed the click operation times, error rates, accuracy deviation, and workload in different target sizes among two layouts (captain and the co-pilot area) and three positions (left/right area of the front instrument panel and central console), which are L1, L2, L3, R1, R2, and R3. In Fig 6, it is shown that the operation times, error rates, accuracy deviations and workload have obvious changes with different sizes. With the increase in size, the operation times decrease; the error rates also decrease, and the error rates no longer change after reaching the 14 mm, except for the position R2. The accuracy deviations increase and the accuracy decreases, the accuracy deviations are increasing sharply after reaching the 14 mm. The workload decreases, except for the size 12 mm and 15mm, with21 mm having the minimum workload. The paper conducted statistical analysis on the data (see Appendix Table 1 in S1 Appendix for detailed statistical data). The results show significant differences in nine sizes, two layouts, and three positions. And the study utilized repeated measures ANOVA analysis to examine the relationship between the main effects of each factor and their interaction effects on each dependent variable.

#### a) Operation time

Repeated measures ANOVA analysis results show that different layouts, positions, and sizes have significant effects on operation times (F(1, 11) = 24.03, p<0.001, partial η^2^ = 0.69; F(2,22) = 21.97, p<0.001, partial η^2^ = 0.67; F(8,4) = 389.43, p<0.001, partial η^2^ = 0.99) (as shown in Appendix Table 2 in S1 Appendix). In addition, a pairwise test was conducted to compare operation times among different sizes (as shown in Table 5). The results indicate that there is no significant difference between the adjacent sizes ranging from 9–21 mm, but there is a significant difference with most other sizes. When the interval is greater than 2 mm, there is a significant difference between sizes 9–11 mm and other sizes. Therefore, it is recommended to use a 3 mm interval in experiments involving small sizes to obtain more noticeable results. When the interval is greater than 3 mm, there is a significant difference between sizes 12, 15, and 18 mm compared to other sizes. Therefore, it is recommended to use a 5–6 mm interval in experiments involving larger sizes to ensure clear and noticeable results. The interval between sizes 18 mm and 21 mm is 3 mm, although the average operation time for 21 mm (ME = 691.30 ms, STD = 12.76) is smaller than that for 18 mm, it is not significantly different from 18 mm (ME = 710.47 ms, STD = 13.71). Combining the statistical data, it shows that the operation time of 21 mm under R3 and R2 is different from that in other positions. There is a slight increase, which may be influenced by the layouts and positions.

**Table 5:** Pairwise test results on operation times between sizes.

P Value	10	11	12	13	14	15	18	21
**9**	>0.05	>0.05	0.045	0.002	0.000	0.001	0.000	0.000
**10**	-	>0.05	>0.05	0.018	0.049	0.002	0.000	0.000
**11**	-	-	>0.05	>0.05	0.030	0.004	0.000	0.000
**12**	-	-	-	>0.05	>0.05	>0.05	0.002	0.000
**13**	-	-	-	-	>0.05	>0.05	>0.05	0.000
**14**	-	-	-	-	-	>0.05	>0.05	0.000
**15**	-	-	-	-	-	-	>0.05	0.044
**18**	-	-	-	-	-	-	-	>0.05

The data analysis of various layouts in the paper also shows that the operation time of the co-pilot area (ME = 800.29 ms, STD = 15.50) is longer than that of the captain area (ME = 753.32 ms, STD = 17.44), and there is a significant difference between the two layouts. The participants used right-handed operation in the captain area, while left- handed operation was used in the co-plot area. Since all the participants are right-handed, their handedness has an impact on the experiment. This is also influenced by the space limitations in the cockpit. In the captain’s area, most screens are located to the right, making right-hand operation more convenient. Conversely, in the co-pilot’s area, most screens are located to the left, making left-hand operation more convenient. Therefore, the paper researches whether the layout would have any effects on operation. According to the average operation times, the co-pilot’s area is slower, but this difference varies among different positions. And the differences between the captain’s and co-pilot’s areas are significant in positions 1, and 2, with an average of 49 ms and 53 ms, respectively. However, in position 3, the difference is only 24 ms. And there are significant differences in operation times among different positions. Position 3 has the shortest operation time (ME = 735.51 ms, STD = 14.93), followed by area position 2 (ME = 794.69 ms, STD = 18.44), and position 1 (ME = 800.21 ms, STD = 17.37) has the slowest operation time. In addition, a pairwise test was conducted between positions (as shown in Appendix Table 3 in S1 Appendix). The results show that there is no difference between positions 1 and 2, but there is a significant difference between positions 1, 2, and 3. There are significant differences between the captain’s area and co-pilot’s area. However, position 3 has the lowest operating time, whether in the captain’s or co-pilot’s area. Therefore, when using the touch screen, the better position is position 3, whether it is in the captain’s area or the co-pilot’s area.

#### b) Error rate

Different layouts and positions have significant effects on the error rates (F(1,11) = 76.23 p<0.001, partial η^2^ = 0.87; F(2,22) = 16.69, p<0.001, partial η^2^ = 0.60) (as shown in Appendix Table 4 in S1 Appendix). There is no significant difference in error rates among different sizes. Therefore, the paper analyzed the layouts and positions, and found that the error rates of the co-pilot area (ME = 0.022) are higher than those in the captain area (ME = 0.009). There are significant differences between the layouts, and the differences among each position are also distinct (as shown in Appendix Table 5 in S1 Appendix). The error rate of position 3 is the lowest (ME = 0.008), followed by position 1 (ME = 0.011), and position 2 has the highest error rate (ME = 0.028). The error rates in the position 2 are relatively high, especially in the co-pilot’s area. The co-pilot’s area utilizes left-hand operation, which also leads to a higher error rate in the R2 area. In addition, the error rates in three positions under the captain’s area is lower than those in the co-pilot’s area, indicating that the difference in error rates could be attributed to handedness.

#### c) Accuracy

Different layouts, positions, and sizes have significant effects on accuracy deviations (F(1,11) = 34.74, p<0.001, partial η^2^ = 0.76; F(2,22) = 26.73, p<0.001, partial η^2^ = 0.71; F(8,88) = 161.03, p<0.001, partial η^2^ = 0.94) (as shown in Appendix Table 6 in S1 Appendix). A pairwise test was conducted to accuracy deviations among different sizes (as shown in Appendix Table 7 in S1 Appendix). There are significant differences between each size, except for sizes 12, 13, and 14 mm. In addition, there are also differences in different layouts and positions (as shown in Appendix Table 8 in S1 Appendix). The accuracy of the captain area (ME = 3.39) is lower than that of the co-pilot area (ME = 3.62). Position 3 has the highest best accuracy (ME = 3.37), followed by position 1 (ME = 3.51), and the worst is position 2 (ME = 3.65). There is a significant difference in accuracy between the captain and co-pilot, indicating that the difference in error rate could be attributed to handedness. And the accuracy deviation in position 2 is relatively high in both the captain’s and co-pilot’s areas. Additionally, the use of left-hand operation in the co-pilot’s area also leads to higher accuracy deviations in the R2 area.

#### D) Workload

**Fig 7:**
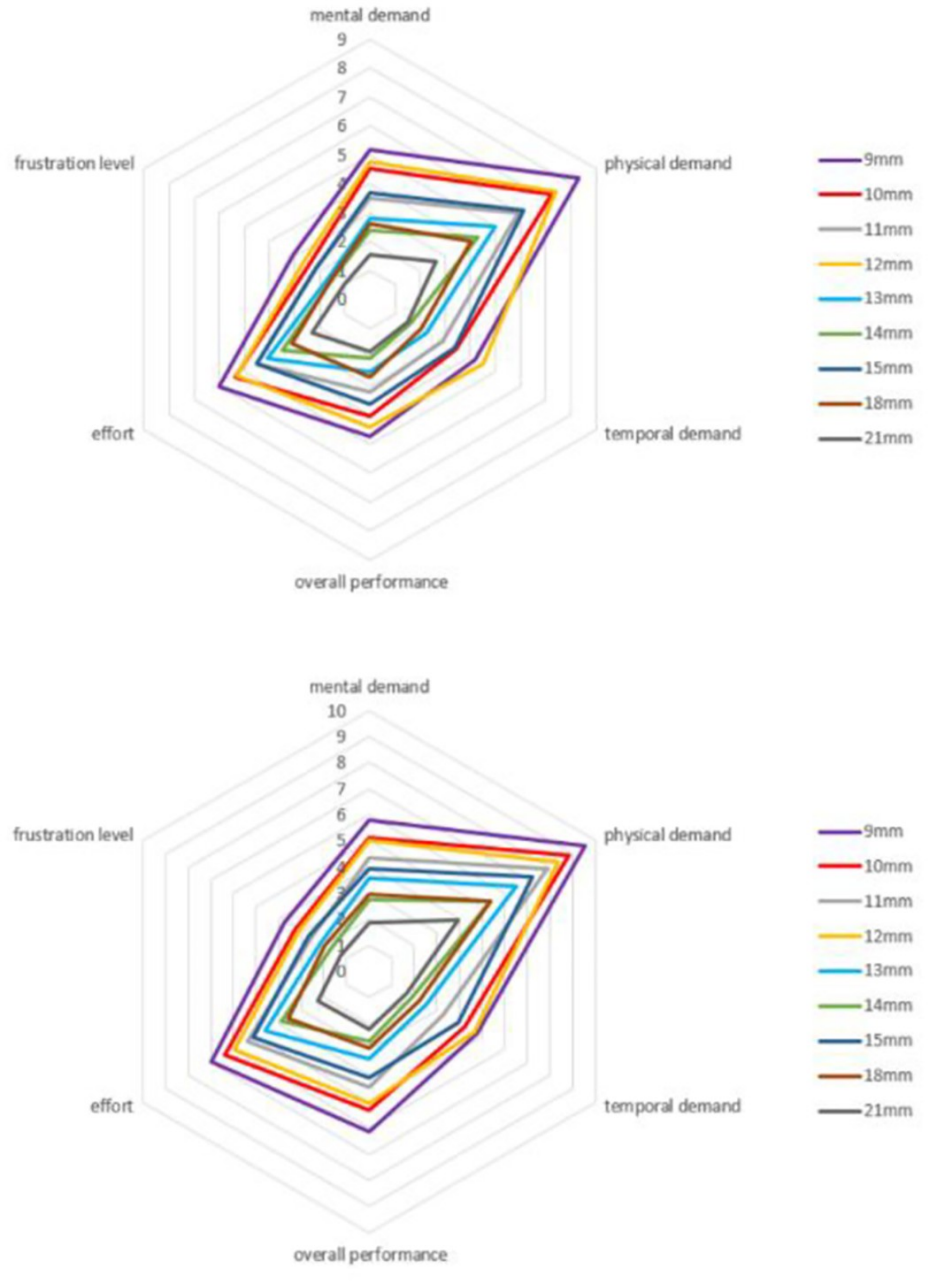
Effects of sizes on workload at Captain area (the upper figure) and co-pilot area (the lower figure).

Different layouts have a significant effect on workload (F(1, 11) = 20.35, p = 0.001, partial η^2^ = 0.65) (as shown in Appendix Table 9 in S1 Appendix). There is no significant difference among sizes. However, it is shown in Fig 6 that as the size increases, the workload continues to decrease, and there are fluctuations in sizes 12 and 15 mm. That is to say, the workload of 12 mm and 15 mm is higher than that of the surrounding sizes. Therefore, the paper analyzed the six dimensions of workload for two layouts (as shown in Fig 7). It can be seen that the workload of 12 mm and 10 mm is similar, with 12 mm being higher in the temporary demand dimension. Additionally, the workload of 15 mm and 11 mm is similar, with15 mm being higher in the temporary demand dimension. Due to the workload being only based on different sizes and layouts, the experiment did not consider the positions. This may be the reason for the variation, and further experimental analysis is needed to determine the specific cause. In addition, the results from six dimensions indicate that physical demand and effort account for a relatively high proportion in the experiment, followed by mental demand and overall performance. In order to reduce workload, on the one hand, we should consider the optimal layouts and sizes; and on the other hand, measures should be taken to reduce physical demand and effort. In addition, the workload of the captain’s area (ME = 3.58) is smaller than that of the co-pilot’s area (ME = 4.26). In all six dimensions, the values in the co-pilot’s area are higher than those in the captain’s area, especially in the physical demand dimension, which is 19% higher. This may be due to the configuration of the right positions and handedness. Therefore, when using the touch screen in the co-pilot’s area, considerations should be given to reducing the workload in terms of physical demand dimension.

### 3.2 Drag experiment

**Fig 8:**
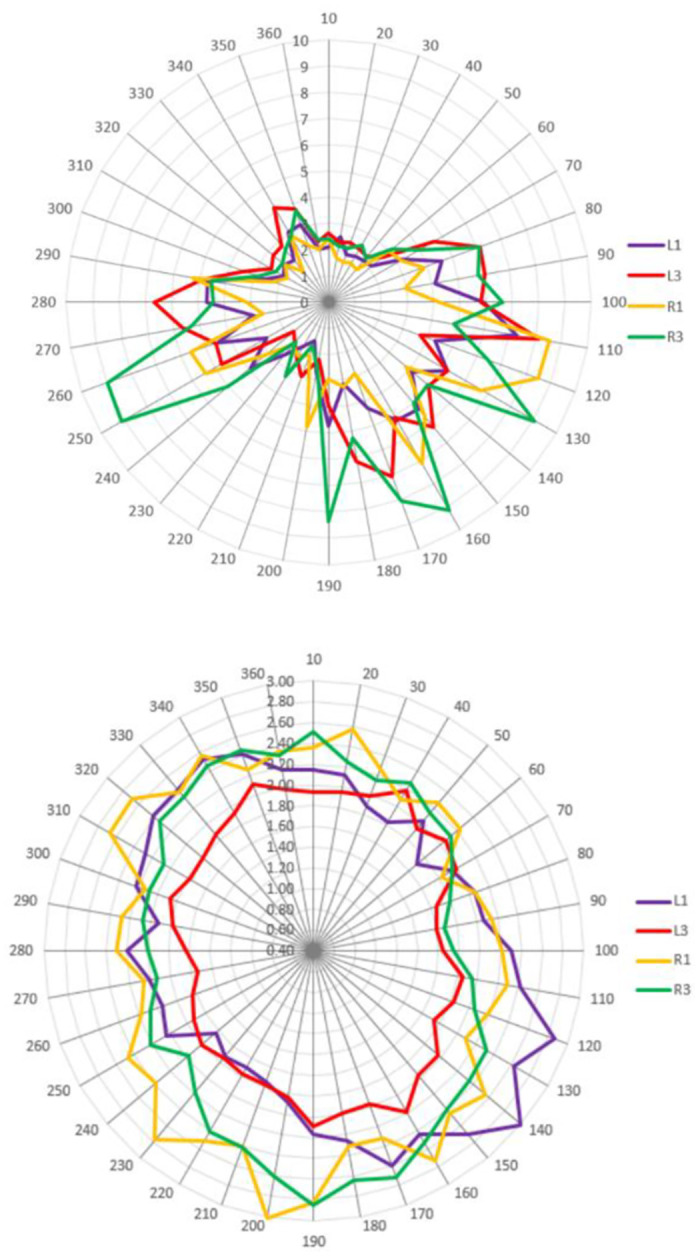
Dragging operation speeds (the upper figure) and accuracy deviations (the lower figure) at different layouts and positions.

The operation speeds of the dragging experiments at different dragging angles are shown in Fig 8. There are local peaks in the dragging operation speeds at 110°, 160°, 250°, and 260°(with the zero direction being true north and increasing clockwise), which have the fastest speeds. On the other hand, at 50°, 210°, 320°, and 330°, the operation speeds reach the local minimum, with the lowest speeds. The average operation speed at each angle is shown Appendix in Table 11 in S1 Appendix. In addition, the operation speed of R3 is higher than that of other positions (as shown in the Fig 8), while its accuracy is lower. The paper conducted a repeated measures ANOVA analysis to examine the relationship between the main effects of each factor and their interaction effects on each dependent variable.

#### a) Operation speed

The results of the repeated measures ANOVA analysis show that different positions and dragging angles have significant effects on operation speeds (F(1, 11) = 8.75, p = 0.013, partial η^2^ = 0.44; F(35, 385) = 11.40, p<0.001, partial η^2^ = 0.51) (as shown in Appendix Table 10 in S1 Appendix). Statistical analysis results of the dragging operation speeds in different angles (as shown in Appendix Table 11 in S1 Appendix) show that the dragging operation speeds at angles 80°-190° and 250°-290° are faster than those at other angles, followed by angle 240°, angles 340°-350°, and the remaining angles are slower. And a pairwise test was conducted on these angles, as shown in Appendix Table 12 in S1 Appendix. The results show that there are significant differences between angles of 100°, 120°, 270°, 290°, and 10°-60°. That is to say, the operation speeds at angles 10°-60° are the slower, while the operation speeds at angles 100°, 120°, 270°, and 290° are faster.

In addition, there are also significant differences in operation speed among different layouts and positions. The operation speed of the captain’s area (ME = 3.76 mm/s) is slower than that of the co-pilot’s area (ME = 4.05 mm/s). The participants in the captain’s area used their right hand, while those in the co-pilot’s area used their left hand. The operation speed of the left hand is actually higher than that of the right hand, which is an interesting discovery. In response to this difference, the analysis was conducted at various positions. The operation speed of position 1 is slower than that of position 3, whether in the captain’s or co-pilot’s area. The operation speeds of positions L1 and R1 are similar, but position R3 is faster than that of L3. In other words, the operation speed in the R3 position is relatively faster, resulting in higher operation speed in the co-pilot’s area than that in the captain’s area. In addition, whether in the captain’s or co-pilot’s area, the operation speed of position 3 (ME = 4.33 mm/s) is faster than that of position 1 (ME = 3.48 mm/s).

#### b) Accuracy

The results of the repeated measures ANOVA analysis show that different layouts, positions, and dragging angles have significant effects on operation accuracy deviations (F(1, 11) = 27.89, p<0.001, partial η^2^ = 0.72; F(1, 11) = 17.44, p = 0.002, partial η^2^ = 0.61; F(35, 385) = 6.03, p<0.001, partial η^2^ = 0.35) (as shown in Appendix Table 13 in S1 Appendix). The statistical data shows that the accuracy of angles between 70°-100° and 260°-270° is higher than other angles, while the lowest accuracy are between 140°-190°. And a pairwise test was conducted on these angles, as shown in Appendix Table 14 in S1 Appendix. The results show that there are significant differences between the 80°-90° and 140°-190° ranges. That is to say, the accuracy of angles between 80°-90° is the highest, while the accuracy of angles between 140°-190° is the lowest. In addition, there are also significant differences among different layouts and positions. The accuracy of the co-pilot’s area is lower than that of the captain’s area, and the accuracy of position 3 is higher than that of position 1. In response to this difference, an analysis was conducted at various positions. The accuracy of position 1 is lower than that of position 3, whether in the captain’s or co-pilot’s area. The accuracy of positions L1 and R1 is similar, but position R3 is lower than that of L3. The high accuracy of position L3 results in a lower accuracy of the co-pilot’s area compared to that of the captain’s area.

#### c) Workload

**Fig 9:**
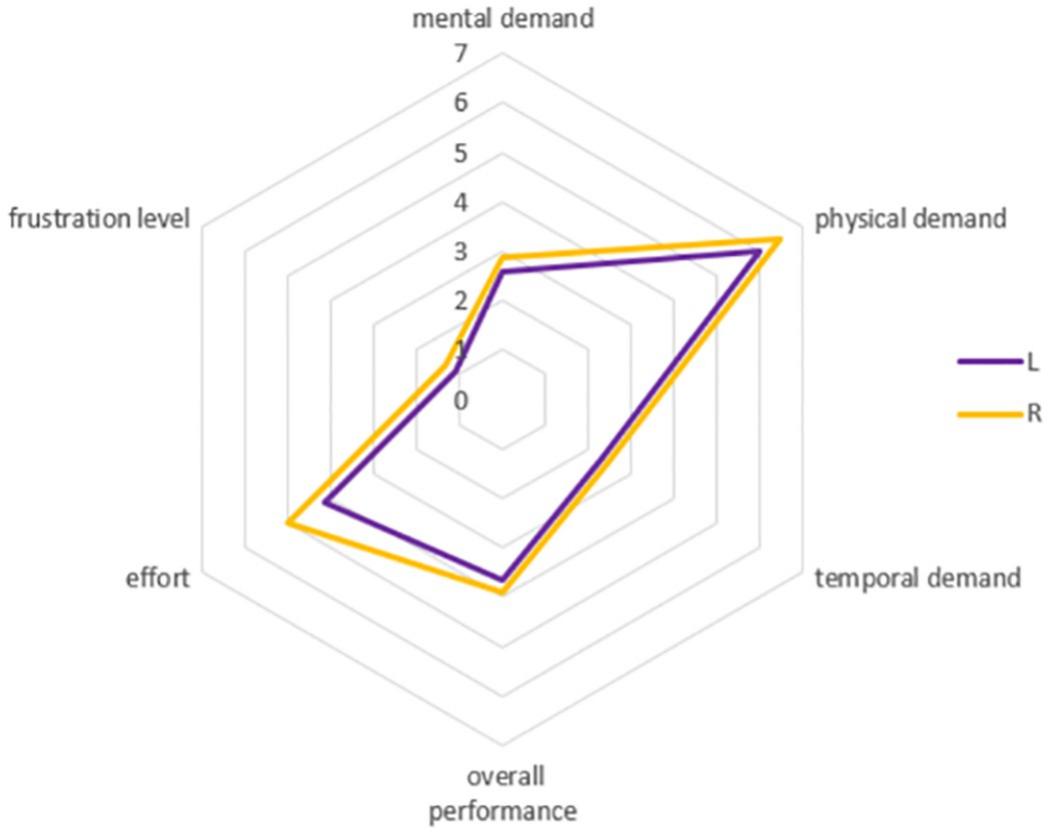
The workload at captain and co-pilot area in dragging experiments.

The workload of the participants was evaluated based on the layout of the captain and co-pilot. There is no significant difference between the two layouts. The captain’s workload is 3.3, which is lower than the co-pilot’s workload of 3.68. In addition, the paper analyzed six dimensions separately, and the results are shown in Fig 9. Among them, the values of physical demand and effort are relatively high. Therefore, measures should mainly focus on these two aspects in order to reduce workload.

### 3.3 Zoom experiment

**Fig 10:**
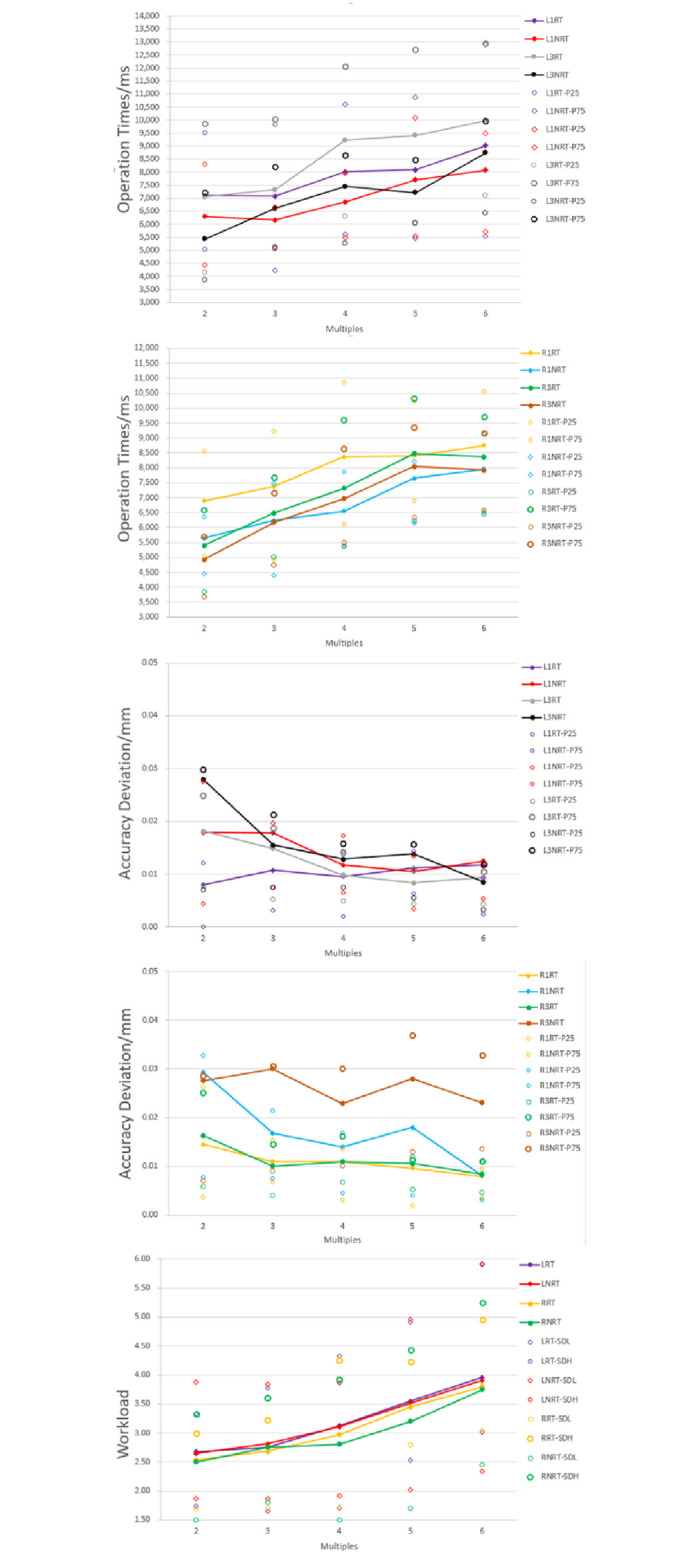
Effects of different positions(L1,L3,R1,R3) and display modes(real time or not real time) on operation performance and workload.

In the zoom in/out experiments, the operation times, accuracy deviations, and workload under different zoom in/out multiples, positions, layouts, and display modes are analyzed respectively. The results are shown in the Fig 10. It shows that as the zoom multiples increase, the operation times also increase. And the operation time with the real-time(RT) display is generally higher than that with the non-real-time(NRT) display. The paper conducted repeated measures ANOVA analysis to examine the relationship between the main effects of each factor and their interaction effects on each dependent variable.

#### a) Operation time

The results of the repeated measures ANOVA analysis on the operation times show that different display modes and multiples have significant effects on operation times(F(1,22) = 15.88, p = 0.001, partial η^2^ = 0.42; F(4,88) = 68.32, p<0.001, partial η^2^ = 0.76) (as shown in Appendix Table 15 in S1 Appendix). There are significant differences among various multiples. The larger the multiples, the more operation times it takes, which aligns with our expectations. In addition, a pairwise test was conducted among multiples (as shown in Appendix Table 16 in S1 Appendix), and the results show significant differences between various multiples, except for multiples 5 and 6. This means that when the multiple increases to a certain extent, the difference in operating time is no longer significant. The statistical data for each multiple is shown in Appendix Table 17 in S1 Appendix. In addition, the experiment results show that there are no significant differences in layout or position. There is no significant difference in operation times between zooming in and zooming out, as well as in the error rates, accuracy, and workload. The display mode has an impact on the operation time. The operation time required for real-time display (ME = 7906.79 ms) is greater than that for non-real-time display (ME = 6931.62 ms), regardless of whether it is in the captain’s or co-pilot’s area and at positions 1 or 3. The difference will be analyzed in the discussion section.

#### b) Accuracy

Different layouts, display modes, and multiples have significant effects on the accuracy deviations (F(1,22) = 6.32, p<0.02, partial η^2^ = 0.22; F(1,22) = 30.68, p<0.001, partial η^2^ = 0.58; F(4,19) = 3.99, p = 0.016, partial η^2^ = 0.46) (as shown in Appendix Table 18 in S1 Appendix). There is a significant difference in accuracy among different multiples. A pairwise test was conducted among multiples (as shown in Appendix Table 19 in S1 Appendix), and the results show significant differences between multiples 2 and the other multiples. However, the difference between the other multiples is not significant. As the multiples increase, the deviations decrease, the accuracy gradually improves, and tends to stabilize after multiple 4. The accuracy of non-real-time display is generally lower than that of real-time display. Although the operation time of non-real-time display is lower, its accuracy has also decreased. Different positions also have an impact on accuracy. The paper compares and analyzes each position and finds that there is a significant difference between R3 and L1, L3. Statistical data shows that the difference may be caused by the data of R3 with non-real-time display mode, which has a lower operation time. However, the deviation of R3 is significantly high than that of other positions, which may be attributed to the R3 position and left-hand operation.

#### c) Workload

**Fig 11:**
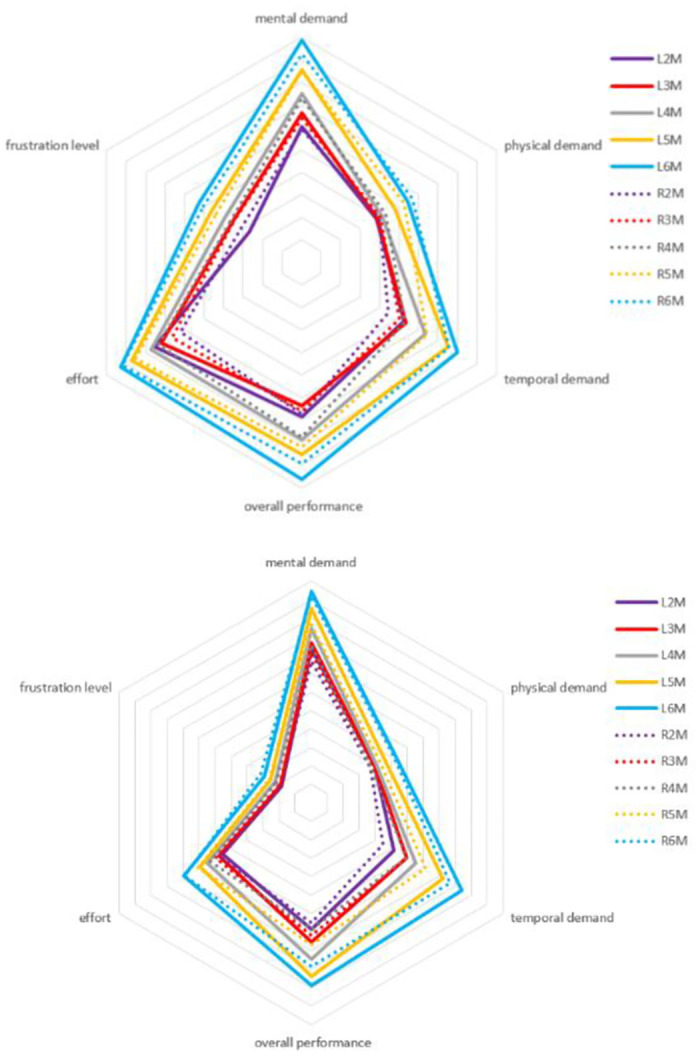
The workload of real-time display (the upper figure) and not real-time display (the lower figure) at different layouts and positions in zoom experiments.

The statistical results show that the larger the zoom multiples, the higher the workload required (F(4,19) = 4.86, p = 0.007, partial η^2^ = 0.51) (as shown in Appendix Table 20 in S1 Appendix). However, there is no significant difference in the workload between different layouts and display modes. The paper analyzed the six dimensions of workload, and the results are shown in Fig 11. Although the display mode does not significantly affect the workload, the frustration level is generally higher in real-time display compared to non-real-time display. Due to the constant need for the operator to monitor and adjust the zoom level in real-time, it can create psychological pressure for the operator. In addition, the workload in the captain’s area is also higher than that in the co-pilot’s area. Based on Fig 10, it can be seen that the workload in the co-pilot’s area is low under the non-real-time display mode, resulting in a reduction in the overall workload of the co-pilot’s area.

## 4. Discussion and conclusion

The experiment analyzes the operation layouts, positions, and sizes of touch screens on click operation performance. The results show that different sizes, layouts, and positions have significant effects on the performance of click operations. And 21 mm size has the minimum operation time and workload, while 18 mm size has the lowest error rate in the clicking tasks. The performance of the captain’s area is generally better than that of the co-pilot. Position 3 is also better than other positions. The difference between the captain’s area and co-pilot’s area may be attributed to handedness. The experimental design takes into account the differences in layouts between the captain’s and co-pilot’s areas. The use of right hand by the captain and the left hand by the co-pilot during the operation may affect the experimental results. This difference can be attributed to the participants’ habitual use of their dominant hand. Additionally, the differences in positions may also contribute to the variations. Therefore, further experimental analysis is needed to study whether there are differences between the participants who use left handedness hands in the co-pilot position. This is needed to address the issue in the future. In addition, the analysis results of experimental data on clicking and dragging show that the operational performance and workload of position 3 are significantly better than those of other positions. Similar findings are also found in [12]. This difference is caused by the layout of position 3. Although position 3 is on the side, its placement angle is around 20°, making it more flexible for participants to operate compared to the front dashboard. Different tilt angles can impact operational performance [14]. In addition, some studies have shown that hand support can impact touch performance [17], and the specific reasons still require further research.

The dragging operation also has many potential applications. For example, the system allows for automatic capture of the waypoints on the map by dragging to create a new flight plan route. It also allows for map movement and page turning of the system page, etc. The paper shows that different dragging direction angles have a significant effect on the performance of the operation, and the angles between 80°-190° and 250°-290° are better. So, in the design process of dragging, the operation activities in the above direction angles, with faster operation speeds and higher accuracy, should be considered a priority.

The most possible application of the zoom operation is the map viewing, which allows for intuitive and quick zooming in/out of a specific area. When checking the terrain, weather radar, TCAS information, and flight plans, it is much more convenient to use the zoom gesture operation. The paper conducted zoom experiments and found that the difference between zooming in and zooming out is not significant. Therefore, it is unnecessary to distinguish whether an operation requires zooming in or zooming out. In addition, the multiples and display modes have significant effects on the performance of the zooming operation. With the increase of zoom multiples, the time required for the operation also increases. Therefore, when the multiple is larger, it is worth considering whether to use the touch screen or directly input the multiple values using touch input. The operation time of real-time display is 14% longer than that of non-real-time display, and its accuracy is 40% higher than that of non-real-time display. In addition, the workload of the real-time display area is about 5% higher than non-real-time display. Due to the participants’ keen focus on the multiple values displayed in real time, the operation time is longer, but the accuracy of the operation is significantly improved. At the same time, the frustration level is higher and the workload is also higher. Therefore, when selecting the zoom control, it is important to consider zoom operations that involve small multiples and do not require high levels of accuracy. If high accuracy requirements are needed, we should consider using direct input of the corresponding multiple to zoom.

The paper also evaluated the workload using the subjective NASA-TLX scale for the click, drag, and zoom experiments. In the click experiment, the workload decreases as the size increases, and in the zoom experiment, the workload increases as the multiple increases. The paper analyzes the six dimensions of workload and finds that the physical demand accounts for a high proportion in the click and drag experiment, while the mental demand is prominent in the zoom experiment. In order to achieve accurate results in the zoom experiment, the participants need more mental demands. Therefore, when we want to reduce workload, both physical and mental demands are the critical factors. For example, adding hand support at the edge of the touch screen can alleviate hand fatigue. Similarly, incorporating visual or operational assistance on the touch screen can reduce the cognitive load required for decision-making.

In addition, the experiment also has some shortcomings and needs to be further improved: 1) the experiment was only conducted with right-hand participants, which may have some impact on the results. The left-hand participant should also be considered, especially in the co-pilot’s area in the future; 2) subjective workload assessment is used in the paper, but some other objective workload measurement methods, such as dual task workload, should be considered; 3) The sample size is reasonable but still small; 4) The current experiments are mainly conducted in the static environment of the cockpit. However, further experiments should be carried out by combining with a vibration environment of an aircraft cockpit, and studying the application of the touch screen in the cockpit further; 5) The experiments select students from aviation schools. Although they have a lot of knowledge about aviation, they do not possess the skills and experience required to operate aviation cockpits. In addition, there are also differences between the characteristics of student participants and those of pilots, flight instructors, or flight trainees. Therefore, further research is needed to determine whether these factors have an impact on experimental operation and workload results. Additionally, the small sample size of the experiment may also affect the experimental results. Therefore, pilots, flight instructors, or flight trainees should be selected for experimental tasks to further explore their impact on touch control applications in future research.

## Supporting information

S1 Appendix (DOCX)

S1 Dataset (XLSX)
