# Supplementary material for: The research of touch screen usability in civil aircraft cockpit
Source: PLoS One. 2024 Feb 8;19(2):e0292849. doi: 10.1371/journal.pone.0292849 (PMC10852311; doi:10.1371/journal.pone.0292849)
Supplement: S1 Appendix — (DOCX) [file pone.0292849.s001.docx]

**Appendix 1**

Drag experiment interfaces are showed in Appendix Fig 1.


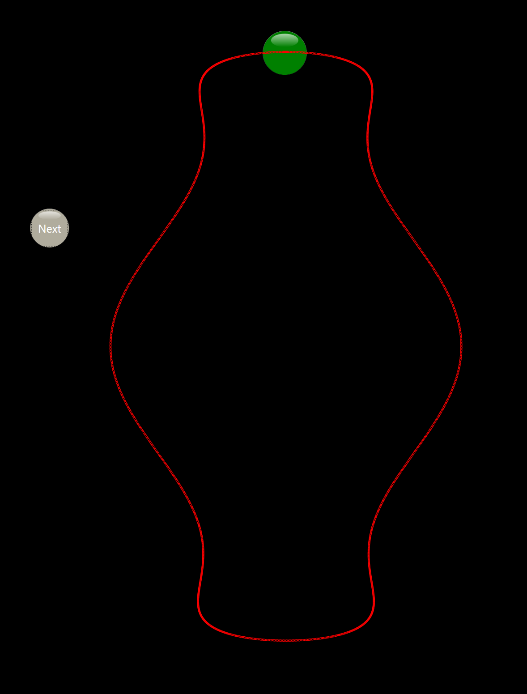

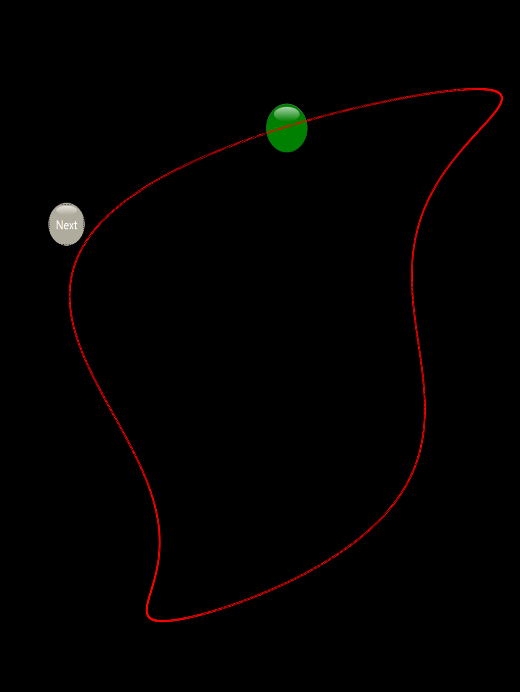

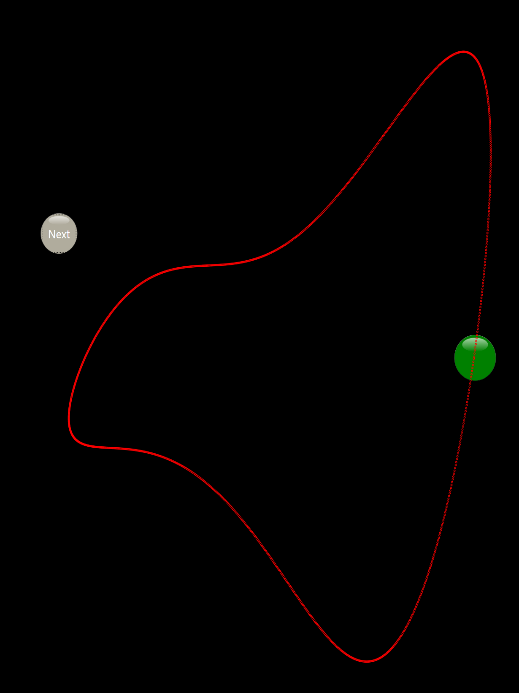

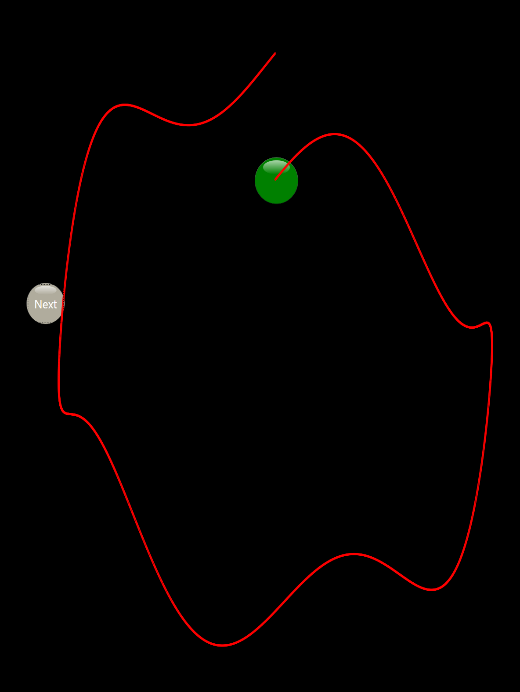

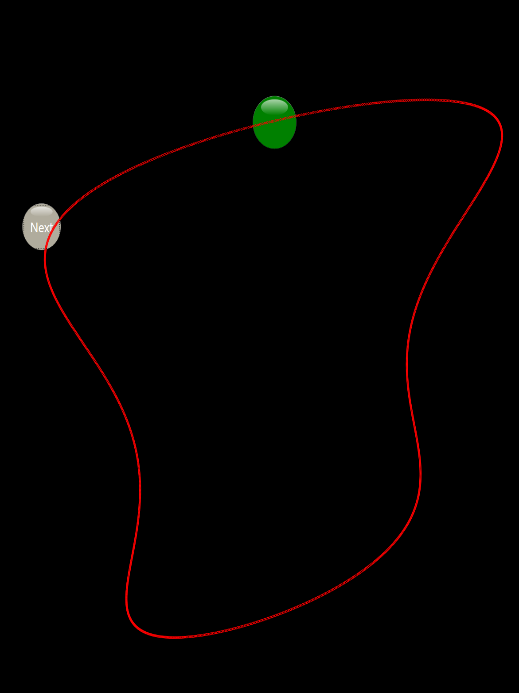

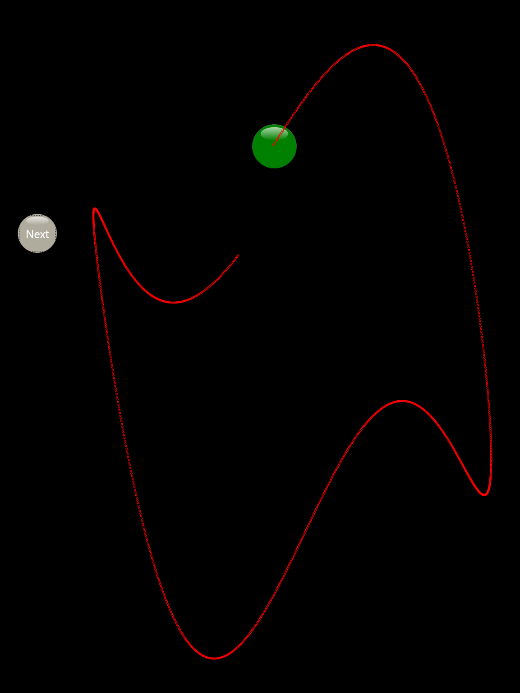

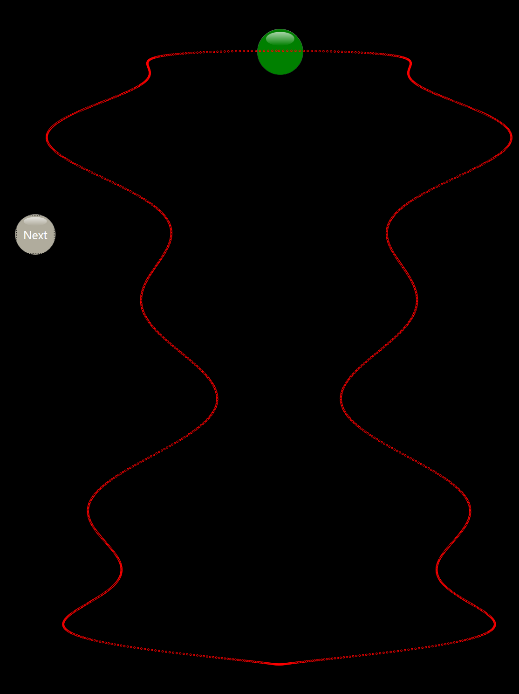

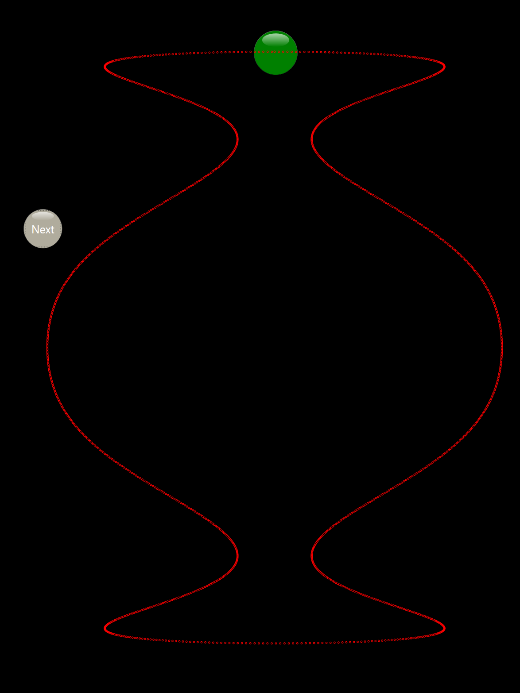

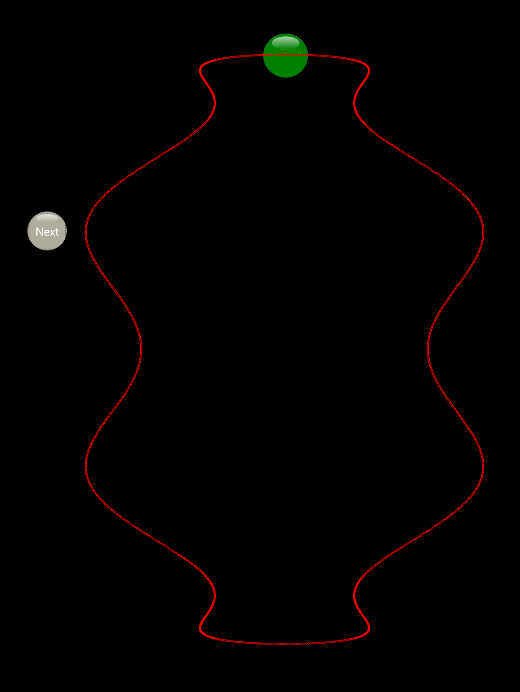

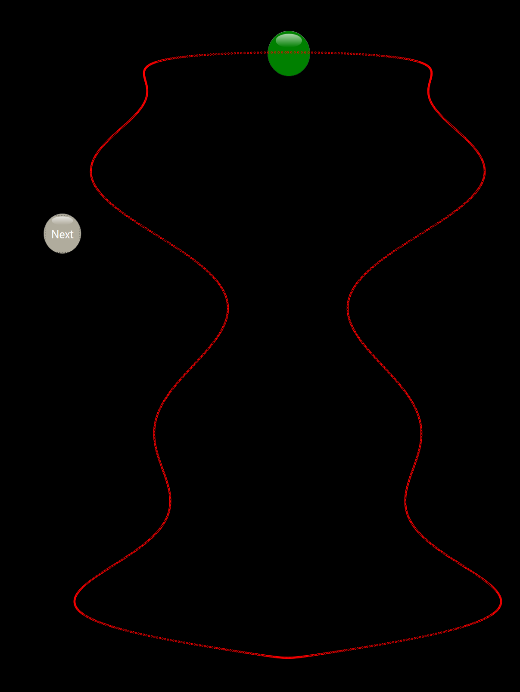


**Appendix Fig 1. Drag experiment interfaces**

**Appendix Table 1. Mean and standard deviation of operation time/error rate/accuracy/workload at different sizes and positions in click experiments**

| **Size/**  **mm** | **Operation Time** | | **Error Rate** | | **Accuracy deviation** | | **Workload** | |
| --- | --- | --- | --- | --- | --- | --- | --- | --- |
|  | Mean/ms | Standard Deviation | Mean | Standard Deviation | Mean/  mm | Standard Deviation | Mean | Standard Deviation |
| **9** | 860.85 | 24.61 | 0.042 | 0.007 | 2.62 | 0.05 | 5.71 | 0.67 |
| **10** | 832.88 | 18.49 | 0.026 | 0.005 | 2.86 | 0.04 | 5.01 | 0.62 |
| **11** | 819.48 | 20.41 | 0.017 | 0.003 | 3.02 | 0.07 | 4.16 | 0.58 |
| **12** | 789.92 | 16.49 | 0.010 | 0.002 | 3.38 | 0.06 | 5.09 | 0.63 |
| **13** | 777.88 | 20.02 | 0.014 | 0.003 | 3.41 | 0.11 | 3.42 | 0.52 |
| **14** | 770.12 | 20.99 | 0.006 | 0.002 | 3.44 | 0.07 | 2.80 | 0.47 |
| **15** | 738.33 | 13.79 | 0.007 | 0.001 | 3.89 | 0.06 | 4.19 | 0.56 |
| **18** | 710.47 | 13.71 | 0.004 | 0.001 | 4.28 | 0.12 | 2.91 | 0.41 |
| **21** | 691.30 | 12.76 | 0.006 | 0.001 | 4.66 | 0.09 | 2.00 | 0.30 |

| **Size/**  **mm** | **Operation Time** | | **Error Rate** | | **Accuracy deviation** | |
| --- | --- | --- | --- | --- | --- | --- |
|  | Mean/ms | Standard Deviation | Mean | Standard Deviation | Mean/  mm | Standard Deviation |
| **L1** | 775.71 | 16.16 | 0.009 | 0.002 | 3.43 | 0.06 |
| **L2** | 760.95 | 21.54 | 0.011 | 0.003 | 3.48 | 0.08 |
| **L3** | 723.31 | 18.30 | 0.005 | 0.001 | 3.26 | 0.06 |
| **R1** | 824.72 | 19.59 | 0.013 | 0.002 | 3.59 | 0.07 |
| **R2** | 828.42 | 18.50 | 0.038 | 0.005 | 3.81 | 0.06 |
| **R3** | 747.71 | 15.32 | 0.011 | 0.002 | 3.48 | 0.10 |

**Appendix Table 2. Repeated measures ANOVA analysis results on operation time in click experiments**

| **Variables** | **F Value** | **P Value** | **Partial η^2^** |
| --- | --- | --- | --- |
| **Layout** | 24.03 | 0.000 | 0.69 |
| **Position** | 21.97 | 0.000 | 0.67 |
| **Size** | 389.43 | 0.000 | 0.999 |
| **Layout*Position** | 3.03 | 0.069 | 0.22 |
| **Layout*Size** | 1.62 | 0.131 | 0.13 |
| **Position*Size** | 0.78 | 0.712 | 0.07 |
| **Layout*Position*Size** | 2.42 | 0.003 | 0.18 |

**Appendix Table 3. Pairwise test results on operation time between positions**

| **P Value** | **2** | **3** |
| --- | --- | --- |
| **1** | >0.05 | 0.000 |
| **2** | — | 0.002 |

**Appendix Table 4. Repeated measures ANOVA analysis results on error rate in click experiments**

| **Variables** | **F Value** | **P Value** | **Partial η^2^** |
| --- | --- | --- | --- |
| **Layout** | 76.23 | 0.000 | 0.87 |
| **Position** | 16.69 | 0.000 | 0.60 |
| **Size** | 4.32 | 0.087 | 0.90 |
| **Layout*Position** | 30.31 | 0.000 | 0.86 |
| **Layout*Size** | 5.47 | 0.001 | 0.33 |
| **Position*Size** | 4.93 | 0.003 | 0.31 |
| **Layout*Position*Size** | 4.22 | 0.003 | 0.28 |

**Appendix Table 5.** **Pairwise test results on error rate between positions in click experiments**

| **P Value** | **2** | **3** |
| --- | --- | --- |
| **1** | 0.003 | >0.05 |
| **2** | — | 0.003 |

**Appendix Table 6. Repeated measures ANOVA analysis results on accuracy deviation in click experiments**

| **Variables** | **F Value** | **P Value** | **Partial η^2^** |
| --- | --- | --- | --- |
| **Layout** | 34.74 | 0.000 | 0.76 |
| **Position** | 26.73 | 0.000 | 0.71 |
| **Size** | 161.03 | 0.000 | 0.94 |
| **Layout*Position** | 3.18 | 0.085 | 0.39 |
| **Layout*Size** | 2.14 | 0.0077** | 0.16 |
| **Position*Size** | 1.70 | 0.051 | 0.13 |
| **Layout*Position*Size** | 2.23 | 0.006 | 0.17 |

****The p value was adjusted according to the method of B/H to control FDR**

**Appendix Table 7.** **Pairwise test results on accuracy deviation between sizes in click experiments**

| **P Value** | **10** | **11** | **12** | **13** | **14** | **15** | **18** | **21** |
| --- | --- | --- | --- | --- | --- | --- | --- | --- |
| **9** | 0.001 | 0.000 | 0.000 | 0.000 | 0.000 | 0.000 | 0.000 | 0.000 |
| **10** | — | >0.05 | 0.000 | 0.002 | 0.000 | 0.000 | 0.000 | 0.000 |
| **11** | — | — | 0.002 | 0.017 | 0.000 | 0.000 | 0.000 | 0.000 |
| **12** | — | — | — | >0.05 | >0.05 | 0.000 | 0.000 | 0.000 |
| **13** | — | — | — | — | >0.05 | 0.010 | 0.000 | 0.000 |
| **14** | — | — | — | — | — | 0.001 | 0.000 | 0.000 |
| **15** | — | — | — | — | — | — | 0.031 | 0.000 |
| **18** | — | — | — | — | — | — | — | >0.05 |

**Appendix Table 8. Pairwise test results on accuracy deviation between positions in click experiments**

| **P Value** | **2** | **3** |
| --- | --- | --- |
| **1** | 0.007 | 0.018 |
| **2** | — | 0.000 |

**Appendix Table 9. Repeated measures ANOVA analysis results on workload in click experiments**

| **Variables** | **F Value** | **P Value** | **Partial η^2^** |
| --- | --- | --- | --- |
| **Layout** | 20.35 | 0.001 | 0.65 |
| **Size** | 5.89 | 0.052 | 0.92 |
| **Layout*Size** | 0.73 | 0.677 | 0.59 |

**Appendix Table 10.** **Repeated measures ANOVA analysis results on operation speed in drag experiments**

| **Variables** | **F Value** | **P Value** | **Partial η^2^** |
| --- | --- | --- | --- |
| **Layout** | 6.37 | 0.055** | 0.37 |
| **Position** | 8.75 | 0.013 | 0.44 |
| **Angle** | 11.40 | 0.000 | 0.51 |
| **Layout*Position** | 2.41 | 0.149 | 0.18 |
| **Layout*Angle** | 2.41 | 0.051 | 0.18 |
| **Position*Angle** | 2.18 | 0.063 | 0.17 |
| **Layout*Position*Angle** | 1.49 | 0.196 | 0.12 |

**** The p value was adjusted according to the method of B/H to control FDR**

**Appendix Table 11. The mean and standard deviation of operation speed in all direction angles**

| **Angle** | **10°** | **20°** | **30°** | **40°** | **50°** | **60°** | **70°** | **80°** | **90°** | **100°** | **110°** | **120°** | **130°** | **140°** | **150°** | **160°** | **170°** | **180°** |
| --- | --- | --- | --- | --- | --- | --- | --- | --- | --- | --- | --- | --- | --- | --- | --- | --- | --- | --- |
| **Mean/mm/s** | 2.39 | 2.17 | 2.03 | 2.13 | 2.03 | 2.74 | 3.75 | 5.17 | 4.73 | 5.64 | 8.05 | 6.49 | 6.56 | 4.48 | 5.56 | 6.64 | 5.58 | 4.46 |
| **Standard Deviation** | 1.08 | 0.79 | 0.74 | 0.73 | 0.64 | 1.75 | 1.97 | 3.10 | 2.66 | 2.63 | 7.28 | 7.23 | 5.82 | 2.26 | 3.86 | 5.46 | 5.46 | 2.82 |
| **Angle** | 190° | 200° | 210° | 220° | 230° | 240° | 250° | 260° | 270° | 280° | 290° | 300° | 310° | 320° | 330° | 340° | 350° | 360° |
| **Mean/mm/s** | 5.00 | 3.07 | 2.12 | 2.51 | 2.18 | 3.45 | 5.50 | 5.90 | 4.10 | 4.70 | 4.86 | 2.79 | 2.17 | 2.36 | 2.24 | 3.27 | 3.23 | 2.22 |
| **Standard Deviation** | 4.71 | 2.29 | 2.63 | 1.92 | 1.21 | 3.26 | 5.24 | 5.29 | 2.31 | 2.67 | 2.65 | 1.41 | 0.80 | 1.00 | 0.99 | 1.87 | 1.47 | 0.62 |

**Appendix Table 12. Pairwise test results on operation speed between direction angles in drag experiments**

| P Value | 90 | 100 | 110 | 120 | 210 | 220 | 230 | 270 | 280 | 290 | 300 | 310 | 320 | 330 | 350 | 360 |
| --- | --- | --- | --- | --- | --- | --- | --- | --- | --- | --- | --- | --- | --- | --- | --- | --- |
| 10 | >0.05 | 0.040 | >0.05 | >0.05 | >0.05 | >0.05 | >0.05 | >0.05 | >0.05 | >0.05 | >0.05 | >0.05 | >0.05 | >0.05 | >0.05 | >0.05 |
| 20 | >0.05 | 0.003 | >0.05 | 0.009 | >0.05 | >0.05 | >0.05 | 0.031 | 0.007 | 0.009 | >0.05 | >0.05 | >0.05 | >0.05 | 0.002 | >0.05 |
| 30 | 0.028 | 0.002 | >0.05 | 0.007 | >0.05 | >0.05 | >0.05 | 0.003 | 0.005 | 0.002 | >0.05 | >0.05 | >0.05 | >0.05 | 0.002 | >0.05 |
| 40 | 0.032 | 0.001 | >0.05 | 0.007 | >0.05 | >0.05 | >0.05 | 0.010 | 0.006 | 0.005 | >0.05 | >0.05 | >0.05 | >0.05 | 0.002 | >0.05 |
| 50 | >0.05 | 0.004 | >0.05 | 0.006 | >0.05 | >0.05 | >0.05 | 0.010 | 0.008 | 0.013 | >0.05 | >0.05 | >0.05 | >0.05 | 0.001 | >0.05 |
| 60 | >0.05 | 0.007 | 0.034 | 0.023 | >0.05 | >0.05 | >0.05 | >0.05 | >0.05 | >0.05 | >0.05 | >0.05 | >0.05 | >0.05 | >0.05 | >0.05 |
| 100 | — | — | >0.05 | >0.05 | >0.05 | 0.012 | 0.004 | >0.05 | >0.05 | >0.05 | 0.009 | 0.003 | 0.031 | 0.006 | >0.05 | 0.007 |
| 120 | — | — | — | — | 0.036 | 0.007 | 0.009 | >0.05 | >0.05 | >0.05 | >0.05 | 0.008 | 0.016 | 0.011 | >0.05 | 0.010 |
| 180 | — | — | — | — | >0.05 | >0.05 | 0.042 | >0.05 | >0.05 | >0.05 | >0.05 | >0.05 | >0.05 | >0.05 | >0.05 | >0.05 |
| 230 | — | — | — | — | — | — | — | >0.05 | 0.005 | 0.020 | >0.05 | >0.05 | >0.05 | >0.05 | 0.007 | >0.05 |
| 270 | — | — | — | — | — | — | — | — | >0.05 | >0.05 | >0.05 | 0.007 | >0.05 | >0.05 | >0.05 | 0.040 |
| 280 | — | — | — | — | — | — | — | — | — | >0.05 | >0.05 | 0.009 | >0.05 | 0.021 | >0.05 | 0.021 |
| 290 | — | — | — | — | — | — | — | — | — | — | 0.018 | 0.009 | >0.05 | 0.031 | >0.05 | 0.035 |
| 310 | — | — | — | — | — | — | — | — | — | — | — | — | >0.05 | >0.05 | 0.005 | >0.05 |
| 330 | — | — | — | — | — | — | — | — | — | — | — | — | — | — | 0.022 | >0.05 |
| 350 | — | — | — | — | — | — | — | — | — | — | — | — | — | — | — | 0.017 |

***Note: The table only shows the results of paired comparisons with significant differences**

**Appendix Table 13.** **Repeated measures ANOVA analysis results on accuracy deviation in drag experiments**

| **Variables** | **F Value** | **P Value** | **Partial η^2^** |
| --- | --- | --- | --- |
| **Layout** | 27.89 | 0.000 | 0.72 |
| **Position** | 17.44 | 0.002 | 0.61 |
| **Angle** | 6.03 | 0.000 | 0.35 |
| **Layout*Position** | 2.91 | 0.116 | 0.21 |
| **Layout*Angle** | 3.82 | 0.000 | 0.26 |
| **Position*Angle** | 2.88 | 0.000 | 0.21 |
| **Layout*Position*Angle** | 3.11 | 0.000 | 0.22 |

**Appendix Table 14. Pairwise test results on accuracy deviation between direction angles in drag experiments**

| P Value | 140 | 150 | 160 | 170 | 180 | 190 | 270 | 300 | 320 | 340 | 360 |
| --- | --- | --- | --- | --- | --- | --- | --- | --- | --- | --- | --- |
| 10 | >0.05 | >0.05 | >0.05 | >0.05 | >0.05 | >0.05 | >0.05 | >0.05 | >0.05 | >0.05 | >0.05 |
| 20 | >0.05 | >0.05 | >0.05 | >0.05 | >0.05 | >0.05 | >0.05 | >0.05 | >0.05 | >0.05 | >0.05 |
| 30 | >0.05 | >0.05 | >0.05 | >0.05 | >0.05 | 0.046 | >0.05 | >0.05 | >0.05 | >0.05 | >0.05 |
| 40 | >0.05 | >0.05 | >0.05 | >0.05 | >0.05 | >0.05 | >0.05 | >0.05 | >0.05 | >0.05 | >0.05 |
| 50 | >0.05 | >0.05 | >0.05 | >0.05 | >0.05 | >0.05 | >0.05 | >0.05 | >0.05 | >0.05 | >0.05 |
| 60 | >0.05 | >0.05 | >0.05 | >0.05 | >0.05 | >0.05 | >0.05 | >0.05 | >0.05 | >0.05 | >0.05 |
| 70 | >0.05 | >0.05 | >0.05 | >0.05 | >0.05 | >0.05 | >0.05 | >0.05 | >0.05 | >0.05 | >0.05 |
| 80 | 0.020 | 0.002 | 0.000 | 0.009 | >0.05 | 0.028 | >0.05 | >0.05 | >0.05 | >0.05 | >0.05 |
| 90 | 0.003 | 0.000 | 0.002 | 0.007 | 0.021 | 0.025 | >0.05 | >0.05 | 0.030 | 0.036 | 0.042 |
| 100 | 0.007 | >0.05 | >0.05 | >0.05 | >0.05 | >0.05 | >0.05 | >0.05 | >0.05 | >0.05 | >0.05 |
| 110 | >0.05 | >0.05 | >0.05 | >0.05 | >0.05 | >0.05 | >0.05 | >0.05 | >0.05 | >0.05 | >0.05 |
| 120 | >0.05 | >0.05 | >0.05 | >0.05 | >0.05 | >0.05 | >0.05 | >0.05 | >0.05 | >0.05 | >0.05 |
| 130 | >0.05 | >0.05 | >0.05 | >0.05 | >0.05 | >0.05 | >0.05 | >0.05 | >0.05 | >0.05 | >0.05 |
| 140 | — | >0.05 | >0.05 | >0.05 | >0.05 | >0.05 | 0.027 | >0.05 | >0.05 | >0.05 | >0.05 |
| 150 | — | — | >0.05 | >0.05 | >0.05 | >0.05 | 0.005 | 0.036 | >0.05 | >0.05 | >0.05 |
| 160 | — | — | — | >0.05 | >0.05 | >0.05 | 0.032 | >0.05 | >0.05 | >0.05 | >0.05 |
| 170 | — | — | — | — | >0.05 | >0.05 | 0.045 | >0.05 | >0.05 | >0.05 | >0.05 |
| 270 | — | — | — | — | — | — | — | >0.05 | >0.05 | >0.05 | 0.048 |

***Note: The table only shows the results of paired comparisons with significant differences**

**Appendix Table 15. Repeated measures ANOVA analysis results on operation time in zoom experiments**

| **Variables** | **F Value** | **P Value** | **Partial η^2^** |
| --- | --- | --- | --- |
| **Layout** | 2.21 | 0.152 | 0.09 |
| **Position** | 0.01 | 0.935 | 0.00 |
| **Display** | 15.88 | 0.001 | 0.42 |
| **Multiple** | 68.32 | 0.000 | 0.76 |
| **Layout * Position** | 3.91 | 0.061 | 0.15 |
| **Layout * Display** | 0.93 | 0.346 | 0.04 |
| **Position * Display** | 0.02 | 0.896 | 0.00 |
| **Layout * Position * Display** | 14.49 | 0.001 | 0.40 |
| **Layout * Multiple** | 3.40 | 0.012 | 0.13 |
| **Position * Multiple** | 4.56 | 0.002 | 0.17 |
| **Layout * Position * Multiple** | 1.30 | 0.307 | 0.21 |
| **Display * Multiple** | 0.72 | 0.564 | 0.03 |
| **Layout * Display * Multiple** | 0.37 | 0.829 | 0.07 |
| **Position * Display * Multiple** | 1.23 | 0.303 | 0.05 |
| **Layout * Position * Display * Multiple** | 0.89 | 0.473 | 0.04 |

**Appendix Table 16. Pairwise test results on operation time between multiples in zoom experiments**

| **Multiple** | **3** | **4** | **5** | **6** |
| --- | --- | --- | --- | --- |
| **2** | 0.021 | 0.000 | 0.000 | 0.000 |
| **3** | — | 0.000 | 0.000 | 0.000 |
| **4** | — | — | 0.013 | 0.000 |
| **5** | — | — | — | 0.180 |

**Appendix Table 17. Mean and standard deviation of operation time/accuracy deviation /workload at different multiples and positions in zoom experiments**

| **Multiple** | **Operation Time** | | **Accuracy deviation** | | **Workload** | |
| --- | --- | --- | --- | --- | --- | --- |
|  | Mean/ms | Standard Deviation | Mean | Standard Deviation | Mean | Standard Deviation |
| **2** | 6094.24 | 377.96 | 0.020 | 0.003 | 2.59 | 0.24 |
| **3** | 6681.50 | 380.48 | 0.016 | 0.002 | 2.75 | 0.26 |
| **4** | 7591.63 | 391.10 | 0.013 | 0.001 | 3.00 | 0.28 |
| **5** | 8126.52 | 397.21 | 0.014 | 0.002 | 3.43 | 0.30 |
| **6** | 8602.14 | 439.47 | 0.011 | 0.001 | 3.85 | 0.35 |

| **Multiple** | **Operation Time** | | **Accuracy deviation** | |
| --- | --- | --- | --- | --- |
|  | Mean/ms | Standard Deviation | Mean | Standard Deviation |
| **L1** | 7439.35 | 476.90 | 0.012 | 0.001 |
| **L3** | 7846.16 | 479.26 | 0.014 | 0.002 |
| **R1** | 7383.01 | 421.54 | 0.014 | 0.002 |
| **R3** | 7008.30 | 340.38 | 0.019 | 0.003 |

**Appendix Table 18. Repeated measures ANOVA analysis results on accuracy deviation in zoom experiments**

| **Variables** | **F Value** | **P Value** | **Partial η^2^** |
| --- | --- | --- | --- |
| **Layout** | 6.32 | 0.020 | 0.22 |
| **Position** | 6.26 | 0.020 | 0.22 |
| **Display** | 30.68 | 0.000 | 0.58 |
| **Multiple** | 3.99 | 0.016 | 0.46 |
| **Layout * Position** | 1.25 | 0.276 | 0.05 |
| **Layout * Display** | 8.48 | 0.008 | 0.28 |
| **Position * Display** | 3.34 | 0.081 | 0.13 |
| **Layout * Position * Display** | 4.77 | 0.075** | 0.18 |
| **Layout * Multiple** | 0.68 | 0.608 | 0.03 |
| **Position * Multiple** | 0.34 | 0.853 | 0.02 |
| **Layout * Position * Multiple** | 4.76 | 0.002 | 0.18 |
| **Display * Multiple** | 2.90 | 0.053** | 0.12 |
| **Layout * Display * Multiple** | 0.51 | 0.727 | 0.10 |
| **Position * Display * Multiple** | 1.18 | 0.327 | 0.05 |
| **Layout * Position * Display * Multiple** | 2.53 | 0.084** | 0.10 |

**** The p value was adjusted according to the method of B/H to control FDR**

**Appendix Table 19. Pairwise test results on accuracy deviation between multiples in zoom experiments**

| **Multiple** | **3** | **4** | **5** | **6** |
| --- | --- | --- | --- | --- |
| **2** | 0.039 | 0.011 | 0.021 | 0.003 |
| **3** | — | >0.05 | >0.05 | >0.05 |
| **4** | — | — | >0.05 | >0.05 |
| **5** | — | — | — | >0.05 |

**Appendix Table 20.** **Repeated measures ANOVA analysis results on workload in zoom experiments**

| **Variables** | **F Value** | **P Value** | **Partial η^2^** |
| --- | --- | --- | --- |
| **Layout** | 2.04 | 0.168 | 0.08 |
| **Display** | 0.08 | 0.777 | 0.00 |
| **Multiple** | 4.86 | 0.007 | 0.51 |
| **Layout * Display** | 0.52 | 0.477 | 0.02 |
| **Layout * Multiple** | 0.98 | 0.443 | 0.17 |
| **Display * Multiple** | 4.27 | 0.012 | 0.47 |
| **Layout * Display * Multiple** | 2.38 | 0.058 | 0.10 |
